# Role of Hakai in m^6^A modification pathway in *Drosophila*

**DOI:** 10.1038/s41467-021-22424-5

**Published:** 2021-04-12

**Authors:** Yanhua Wang, Lifeng Zhang, Hang Ren, Lijuan Ma, Jian Guo, Decai Mao, Zhongwen Lu, Lijun Lu, Dong Yan

**Affiliations:** 1 CAS Key Laboratory of Insect Developmental and Evolutionary Biology, CAS Center for Excellence in Molecular Plant Sciences, Institute of Plant Physiology and Ecology, Chinese Academy of Sciences, Shanghai, China; 2 CAS Center for Excellence in Biotic Interactions, University of Chinese Academy of Sciences, Beijing, China; 3 Gene Regulatory Lab, School of Medicine, Tsinghua University, Beijing, China; 4 State Key Laboratory of Genetic Engineering, School of Life Sciences, Fudan University, Shanghai, China

**Keywords:** Development, Epigenetics, RNA splicing, RNA modification

## Abstract

N6-methyladenosine (m^6^A), the most abundant internal modification in eukaryotic mRNA, is installed by a multi-component writer complex; however, the exact roles of each component remain poorly understood. Here we show that a potential E3 ubiquitin ligase Hakai colocalizes and interacts with other m^6^A writer components, and *Hakai* mutants exhibit typical m^6^A pathway defects in *Drosophila*, such as lowered m^6^A levels in mRNA, aberrant *Sxl* alternative splicing, wing and behavior defects. Hakai, Vir, Fl(2)d and Flacc form a stable complex, and disruption of either Hakai, Vir or Fl(2)d led to the degradation of the other three components. Furthermore, MeRIP-seq indicates that the effective m^6^A modification is mostly distributed in 5’ UTRs in *Drosophila*, in contrast to the mammalian system. Interestingly, we demonstrate that m^6^A modification is deposited onto the *Sxl* mRNA in a sex-specific fashion, which depends on the m^6^A writer. Together, our work not only advances the understanding of mechanism and regulation of the m^6^A writer complex, but also provides insights into how Sxl cooperate with the m^6^A pathway to control its own splicing.

## Abstract (web summary)

Drosophila m6A writer complex regulates alternative splicing of the *Sex-lethal* gene. Here the authors show that a potential E3 ligase Hakai interacts with the fly m6A writer complex and that m6A level is reduced in Hakai mutant flies.

## Introduction

There are a variety of chemical modifications on biological macromolecules, such as proteins, nucleic acids, and glycolipids. Like DNA methylation and histone modification, RNA modification represents an extra layer of epigenetic regulatory mechanism^1,2^. More than 150 chemical modifications in RNA have been discovered, and their biological functions are only starting to be revealed^3^. Chemical modifications of RNA exist in all organisms and for all forms of RNA, including tRNA, rRNA, mRNA, and long noncoding RNA. Common RNA modifications include N6-methyladenosine (m^6^A), N6,2’-O-dimethyladenosine (m^6^A_m_), N1-methyladenosine (m^1^A), 5-methylcytidine (m^5^C), N4-acetylcytidine (ac^4^C), 7-methylguanosine (m^7^G), and pseudouridine (Ψ), etc^4,5^. Among them, m^6^A is the most abundant internal modification of mRNA in eukaryotes. Although m^6^A in mRNA was found more than 40 years ago^6,7^, it was only recently that the field has made extensive progress owing to technological and experimental breakthroughs. By combining m^6^A-specific antibody and high-throughput sequencing, MeRIP-Seq or m^6^A-Seq allows the m^6^A mapping at the whole transcriptome level, thereby providing the possibility to correlate RNA modifications with their biological functions^8,9^. These and subsequent studies revealed that m^6^A sites contain a consensus motif RRACH (R = G/A; H = U/A/C), and m^6^A peaks are enriched in the 3′ untranslated region (UTR) and near the stop codon in yeast and mammals^8–10^. In *Arabidopsis*, m^6^A is enriched not only in 3′UTRs and near the stop codon but also in 5′UTRs and around the start codon^11^. In mammalian cells, m^6^A also accumulates in the 5′UTR region in response to stress conditions such as heat shock^12,13^. The distribution of m^6^A is important since it implies the mechanism by which m^6^A modification regulates its mRNA.

Another major breakthrough is the gradual elucidation of the m^6^A modification pathway by biochemical and genetic studies. The m^6^A is deposited by a multicomponent methyltransferase complex (“writers”)^14–16^, mainly recognized by YTH domain-containing “readers”^17^, and can be removed by FTO and ALKBH5 “erasers”^18,19^, although FTO was also indicated as an m^6^A_m_ demethylase^20^. The key catalytic component of the m^6^A writer complex, Mettl3, was purified and cloned in 1990s^21,22^. Since then, studies from yeast, *Arabidopsis*, *Drosophila*, and mammalian cells have identified several core components of the writer complex^23,24^, including Mettl14^25,26^, WTAP (Fl(2)d)^27–29^, VIRMA (Virilizer)^30,31^, RBM15/15B (Spenito)^32,33^, ZC3H13 (Flacc or Xio)^34–36^, and Hakai^37^. Interestingly, Fl(2)d^38^, Virilizer (Vir)^39^, Spenito (Nito)^40^, and Xio^36^ were first identified from *Drosophila* sex determination screens and later realized as part of the writer complex. They regulate *Drosophila* sex determination by controlling the alternative splicing of the master regulatory gene *Sex-lethal* (*Sxl*)^41–46^. Recently, Mettl3, Mettl14, as well as the reader Ythdc1, were also shown to be involved in this process^33,47,48^. However, the detailed mechanism of how the m^6^A modification cooperates with Sxl protein to modulate its own splicing is still unclear. Thus, *Drosophila* can serve as a unique system to screen components in the m^6^A pathway and pinpoints a critical role for m^6^A in regulating splicing. Other than *Sxl* splicing, *Drosophila* m^6^A genes are highly expressed in the nervous system and exhibit similar wing and behavior defects when mutated^33,36,47,48^. Mutants of several fly m^6^A factors are viable and thus provide an ideal model to study other processes, such as metabolism and immunity, in the future.

Hakai, also known as CBLL1, was found as an interacting protein with several m^6^A writer components in proteomic studies^23,31,49^. It encodes a RING finger-type E3 ubiquitin ligase and was originally identified as an E-cadherin-binding protein in human cell lines^50^. It was proposed that Hakai ubiquitinates E-cadherin at the plasma membrane and induces its endocytosis, thus playing a negative role post-translationally. Due to the key role of E-cadherin in tumor metastasis, especially epithelial–mesenchymal transition, Hakai has been extensively studied mainly using cell culture and overexpression system^51^, but a previous study using the *Drosophila* model did not observe an increase of E-cadherin level in *Hakai* mutants^52^. In *Arabidopsis*, *Hakai* mutants show partially reduced m^6^A levels and the mutant phenotypes are weaker than other writer components^37^. Importantly, the in vivo role of Hakai as a core m^6^A writer component has not been studied in any animal species. Here, we analyzed the role of Hakai in the *Drosophila* m^6^A modification pathway. Our results demonstrated that Hakai is a bona fide member of the m^6^A writer complex, with its mutants showing reduced global m^6^A levels, typical m^6^A mutant phenotypes, and commonly-regulated gene sets. We also obtained a high-quality fly m^6^A methylome using stringent MeRIP-seq, discovered a female-specific m^6^A methylation pattern for *Sxl* mRNA, characterized the role of Hakai in the m^6^A writer complex, and finally revisited the function of Hakai in E-cadherin regulation.

## Results

### Hakai interacts and colocalizes with known m^6^A writer complex subunits

**Fig. 1:**
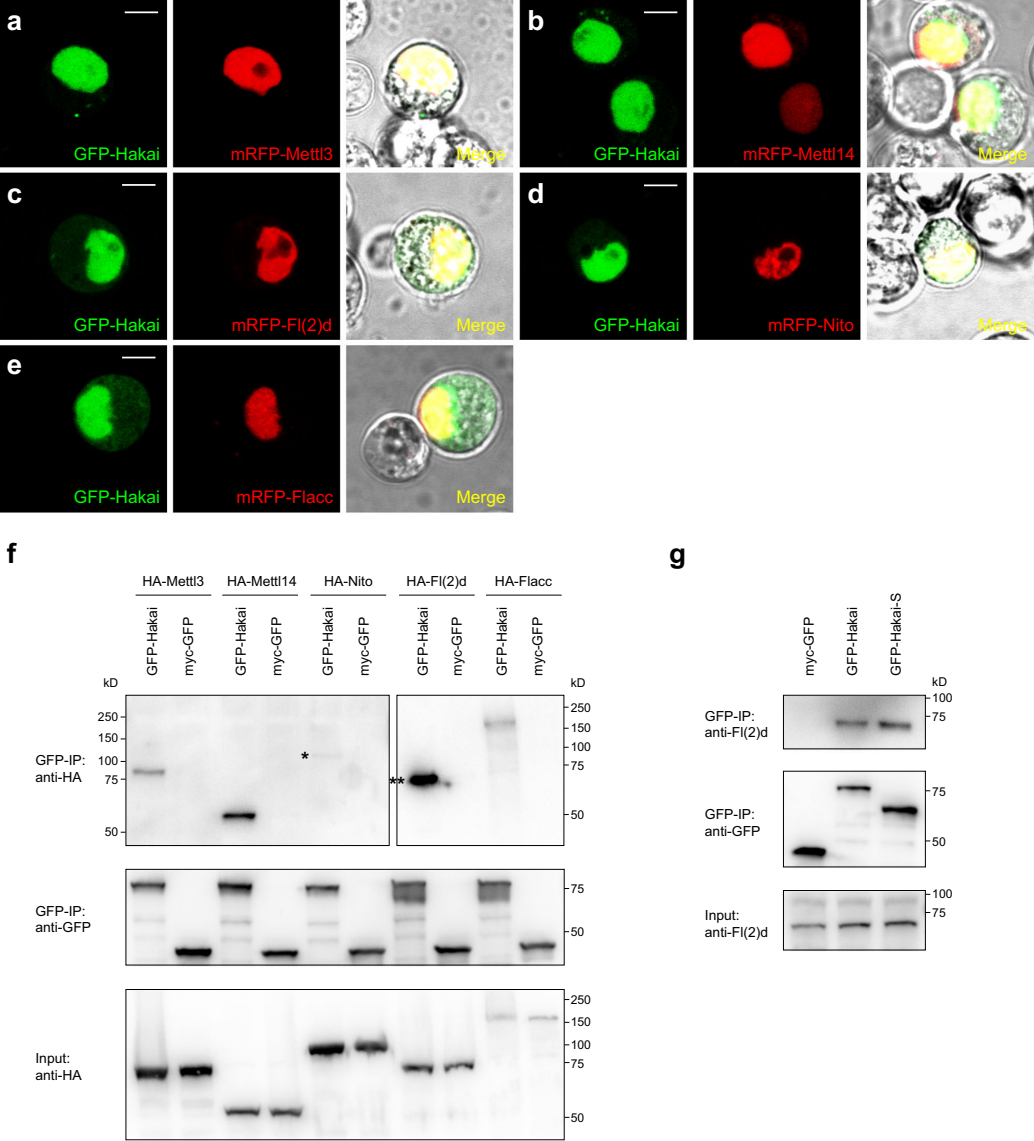
Hakai colocalizes and interacts with other m^6^A writer components. **a**–**e** GFP-Hakai and mRFP-Mettl3, mRFP-Mettl14, mRFP-Fl(2)d, mRFP-Nito, or mRFP-Flacc were co-transfected into S2 cells and their subcellular localization examined in live conditions. All the proteins were predominantly nuclear and GFP-Hakai showed strong co-localization with other factors. Scale bars: 5 μm. **f** GFP-Hakai or myc-GFP and HA-Mettl3, HA-Mettl14, HA-Nito, HA-Fl(2)d, HA-Flacc were co-transfected into S2 cells. Cell lysates were immunoprecipitated using GFP nanobody and analyzed by western blot. myc-GFP was used as a control. GFP-Hakai can pull down HA-Mettl3, HA-Mettl14, HA-Nito, HA-Fl(2)d, and HA-Flacc. Note that much more HA-Fl(2)d was co-IPed than other factors (double asterisk), while the interaction with Nito was the weakest (asterisk). **g** myc-GFP, GFP-Hakai, or GFP-Hakai-S (short isoform) were transfected into S2 cells. Cell lysates were immunoprecipitated using GFP nanobody and analyzed by western blot. GFP-Hakai and GFP-Hakai-S can pull down endogenous Fl(2)d protein to a similar level. Source data are provided as a Source Data file. The experiments in **a**–**g** were repeated at least twice independently with similar results.

**Fig. 2:**
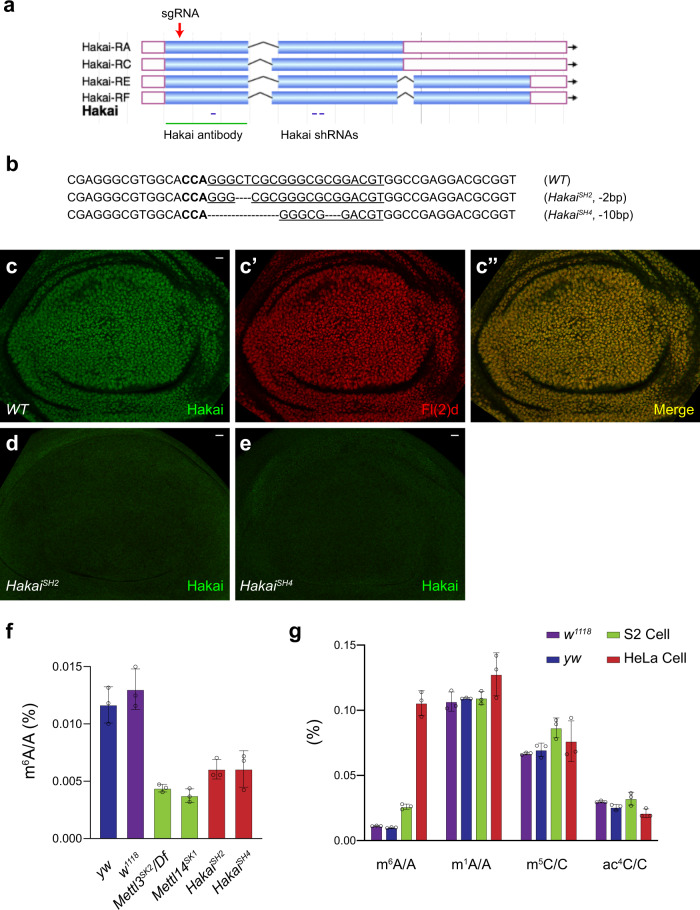
Hakai is required to maintain proper levels of m^6^A methylation. **a** Flybase JBrowse view of the *Hakai* gene locus. *Hakai* has four transcripts due to alternative splicing that generates two long and two short protein isoforms. The positions of three independent shRNAs, *Hakai* sgRNA, and the protein region used to generate a Hakai antibody are indicated. **b** Sequencing results showing frameshift indels in *Hakai*^*SH2*^ and *Hakai*^*SH4*^ flies generated by CRISPR/Cas9-mediated mutagenesis. The targeted genomic DNA sequence is underlined and the NGG PAM sequence is in bold type. **c**–**c”** Hakai and Fl(2)d antibody staining in WT wing discs showing a high degree of co-localization. Hakai antibody staining was strongly reduced in *Hakai*^*SH2*^ (**d**) or *Hakai*^*SH4*^ (**e**) homozygous mutant wing discs. The experiments in **c**–**e** were repeated at least twice independently with similar results, and each time around 30 wing discs for any genotype were examined. Scale bars: 10 μm. **f** Quantifications of m^6^A relative to A in mRNA extracted from male adult flies by LC-MS. Compared to *yw* and *w*^*1118*^ controls, m^6^A levels dropped to about 30% in *Mettl3* or *Mettl14* mutants and to <50% in *Hakai*^*SH2*^ or *Hakai*^*SH4*^ mutants. **g** Quantifications of m^6^A/A, m^1^A/A, m^5^C/C, and ac^4^C/C levels in mRNA extracted from *yw* and *w*^*1118*^ male flies, *Drosophila* S2 cells, and human HeLa cells. Note the substantial difference between fly and human m^6^A levels. **f**, **g** Data are presented as mean ± SD from three biological replicates. Source data are provided as a Source Data file.

Since Hakai was found as an interacting protein with other m^6^A writer components in the mammalian proteomic study^23^, we searched the large-scale *Drosophila* Protein interaction Map (DPiM) database^53^. Hakai as bait can pull down Fl(2)d, Vir, and Nito in affinity purification and mass spectrometry experiments; on the other hand, Flacc as a bait can pull down Hakai (Supplementary Fig. 1b). Similarly, our own previous mass-spec study using Fl(2)d or Nito as bait can reciprocally pull down Hakai (Supplementary Fig. 1a)^36^. To confirm these interactions, we performed both co-localization and co-immunoprecipitation (Co-IP) assays. GFP-Hakai localized to nucleus in live S2 cells and co-localized well with mRFP-Mettl3, mRFP-Mettl14, mRFP-Fl(2)d, mRFP-Nito, and mRFP-Flacc (Fig. 1a–e). Next, we transfected GFP-Hakai and different HA-tagged constructs in S2 cells, and used myc-GFP as a control. In the Co-IP experiments, GFP-Hakai, but not myc-GFP, was able to pull down HA-Mettl3, HA-Mettl14, HA-Nito, HA-Fl(2)d, and HA-Flacc (Fig. 1f). Interestingly, the pulldown between Hakai and Fl(2)d was particularly strong compared to other factors, suggesting that Hakai may directly interact with Fl(2)d, while the interaction between Hakai and Nito was the weakest (Fig. 1f). We then further examined the ability of GFP-Hakai to pull down endogenous Fl(2)d proteins using the available monoclonal antibody^42^. *Hakai* transcript is alternatively spliced, producing long and short protein isoforms (Fig. 2a), both of which contain a C3HC4 RING finger domain and a C2H2 zinc-finger domain (Supplementary Fig. 2a). We also included the short isoform in our assay and found that both GFP-Hakai (long isoform) and GFP-Hakai-S (short isoform) can robustly pull down Fl(2)d to a similar extent (Fig. 1g). Together, these data suggest that Hakai is a conserved core component of the m^6^A writer complex and its N-terminal domains are important for its interaction.

### Hakai is required to maintain proper levels of m^6^A methylation

*Hakai* transcript shows a similar expression pattern to those of other m^6^A writers and readers^36^, with high expression in the CNS, ovary, fat body and imaginal discs (Supplementary Fig. 2b; modENCODE developmental and tissue expression database^54^). During development, its expression is high in early embryos, decreases during larval stages, and rises again at pupal stages (Supplementary Fig. 2b), which coincides with the reported m^6^A levels^33^. To monitor endogenous protein expression, we raised an antibody against Hakai (Fig. 2a) and found that it is a ubiquitously-expressed nuclear protein that strongly colocalizes with Fl(2)d (Fig. 2c–c”). To further investigate Hakai function, we generated a sgRNA and constructed or obtained three nonoverlapping shRNA lines (Fig. 2a)^55,56^. By crossing the *U6:3-Hakai sgRNA* with *nanos-Cas9*^57^, we generated a series of *Hakai* mutants with various small deletions and/or insertions. We chose two alleles, *Hakai*^*SH2*^ and *Hakai*^*SH4*^, for further analysis, since they represent different early frameshift mutations that are expected to disrupt translation (Fig. 2b). Indeed, only the background level of Hakai antibody staining remained in *Hakai* homozygous mutant wing discs compared to wild-type (Fig. 2d, e, compared to c), suggesting these are null or strong loss-of-function alleles. *Hakai* homozygous mutants are semi-lethal and only produce viable adult flies in noncrowded conditions, which have delayed developmental time, smaller size, and reduced lifespan.

We then measured N6-methyladenosine levels in *Hakai* mutant adults by quantitative liquid chromatography–mass spectrometry (LC-MS) and used *yw* and *w*^*1118*^ as wild-type controls. We used an external calibration curve prepared with A and m^6^A standards to determine the absolute quantities of each ribonucleoside (Supplementary Fig. 3). After two rounds of polyA selection, m^6^A levels dropped to around 30% in *Mettl3* or *Mettl14* mutant flies, while m^6^A levels reduced more than half in *Hakai*^*SH2*^ or *Hakai*^*SH4*^ mutants (Fig. 2f). These results clearly indicate a critical role of Hakai in m^6^A methylation. During our analysis, we found that the m^6^A levels we measured in *Drosophila* (0.01–0.02% of adenosine after two rounds of polyA purification) were one magnitude lower than those in mammals (0.1–0.4% of adenosine)^1^. This result is consistent with a previous study showing that m^6^A accounts for 0.04% of adenosine after one round of polyA selection in *yw* flies^47^. Thus, we further measured the m^6^A level, as well as the level of several other RNA modifications such as m^1^A, m^5^C, and ac^4^C, in *w*^*1118*^, *yw* flies, S2 cells, and human HeLa cells. To our surprise, m^6^A level was five to ten times higher in human cells than those in *Drosophila*, while m^1^A, m^5^C, and ac^4^C levels were comparable (Fig. 2g). These results imply that the function and mechanism of the m^6^A pathway may be quite different between human and fly.

### *Sxl* alternative splicing and adult fly behavior were defective in *Hakai* mutant

**Fig. 3:**
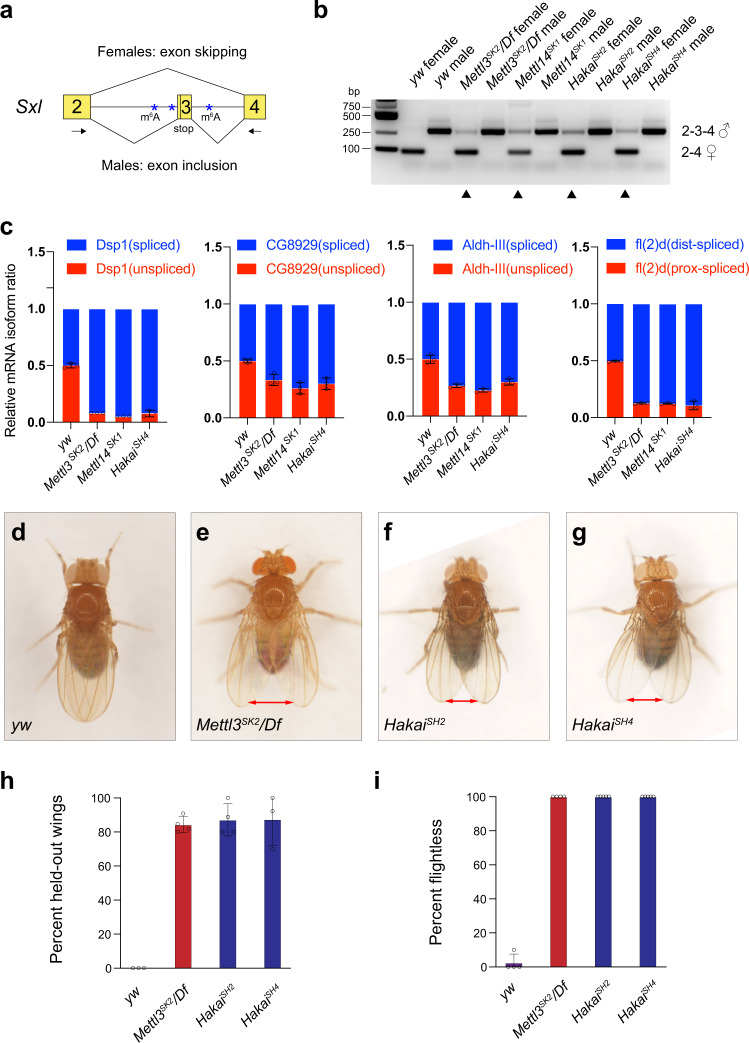
Hakai regulates *Sxl* alternative splicing and adult fly behavior. **a** Diagram showing the alternative splicing event that produces male- or female-specific *Sxl* transcripts. The arrows indicate primers used for RT-PCR. **b** *Sxl* splicing was analyzed by RT-PCR using RNAs extracted from adult flies of indicated genotypes. Note the appearance of male-specific bands in *Mettl3*^*SK2*^*/Df*, *Mettl14*^*SK1*^, *Hakai*^*SH2*^, *Hakai*^*SH4*^ females (arrowheads). Male-specific bands: 2–3–4. Female-specific bands: 2–4. **c** Relative isoform quantification for *Dsp1*, *CG8929*, *Aldh-III* and *fl(2)d* by qPCR in control or m^6^A mutant flies. Hakai is involved in m^6^A-dependent splicing events. Data are presented as mean ± SD from three biological replicates. **d** *yw* flies have their wings properly folded. (**e**) *Mettl3*^*SK2*^*/Df*, (**f**) *Hakai*^*SH2*^, (**g**) *Hakai*^*SH4*^ flies cannot fold their wings and exhibit a held-out wing phenotype (marked by the double arrows). The frequency of flies showing held-out wings were quantified in (**h**). **i** Flies of the indicated genotypes were tested for their flight abilities, and the number of flightless flies was quantified. All flies used from (**c**) to (**i**) were males. **h**–**i** Data are presented as mean ± SD from three to five biologically independent groups (**h**, *n* = 3, 4, 4, 3; **i**, *n* = 4, 4, 5, 5). Source data are provided as a Source Data file.

In *Drosophila*, *Sxl* pre-mRNA is the best-characterized example of m^6^A-modified transcripts. *Sxl* transcripts are alternatively spliced. While the male form includes exon3 that contains a stop codon and leads to early termination of Sxl protein translation, the female form skips exon3 and thus produces a functional Sxl protein (Fig. 3a)^58^. Previously multiple m^6^A sites have been mapped in introns on both sides of exon3 and these modifications were proposed to facilitate the alternative splicing of *Sxl* in female flies^47,48^. Indeed, the switch from female form to male form of *Sxl* splicing occurs in all m^6^A mutants, including *Mettl3*, *Mettl14*, *Ythdc1*, *Fl(2)d*, *Vir*, *Nito*, *Flacc*, thus representing a gold standard to validate components in this pathway^33,34,36,38,41,43,47,48^. To monitor *Sxl* splicing pattern, we used a pair of primers flanking exon3 that detects the small female and large male spliced *Sxl* products in RT-PCR (Fig. 3a, b, lanes 1, 2)^59^. As positive controls, *Sxl* splicing was partially shifted from the female form to the male form in *Mettl3* or *Mettl14* mutant females (Fig. 3b, lanes 3–6, arrowheads). In *Hakai*^*SH2*^ or *Hakai*^*SH4*^ female flies, a large band corresponding to the male-specific spliced form was clearly detected (Fig. 3b, last 4 lanes, arrowheads), similar to *Mettl3* or *Mettl14* mutants.

Disruption of several m^6^A components Fl(2)d, Vir, Nito or Flacc leads to not only aberrant *Sxl* splicing but also strongly reduced Sxl protein levels, thus generating a striking female-to-male transformation phenotype in adult flies^34,36,38,41,43,60^. Mutation of other factors *Mettl3*, *Mettl14*, or *Ythdc1* alone does not affect Sxl protein levels and does not exhibit the transformation phenotype^33,47,48^. We found that Sxl protein level was not reduced in *Hakai*^*SH2*^ or *Hakai*^*SH4*^ female discs (Supplementary Fig. 4a–d). Furthermore, we expressed three *Hakai* RNAi using *dome-Gal4* and did not observe any transformation phenotype in females, as evidenced before^36,41^.

Other than *Sxl*, it was reported that splicing of several other genes, including *Dsp1*, *CG8929*, *Aldh-III*, and *fl(2)d*, depends on the m^6^A pathway^33,34^. We then analyzed the splicing isoforms for these transcripts by RT-qPCR. In *Hakai*^*SH4*^ mutants, the splicing patterns for all four genes were affected similarly to those in *Mettl3* or *Mettl14* mutants (Fig. 3c, see Supplementary Fig. 9f–i for positions of primers used). It is worth noting that in all four cases, the spliced isoforms were increased while the unspliced forms were reduced in m^6^A pathway mutants.

Besides sex determination, m^6^A writer and reader mutants show characteristic adult defects. The most prominent ones are the held-out wings and flightless phenotypes in *Mettl3*, *Mettl14*, *Ythdc1*, or *Flacc* mutants^36,47,48^, likely due to loss of m^6^A functions in the nervous system^33^. Wild-type flies normally keep their wings in a folded position (Fig. 3d, h), however, the majority of *Mettl3* mutant flies cannot fold their wings correctly and exhibit held-out wings (Fig. 3e, h), and 100% *Mettl3* mutant flies cannot fly (Fig. 3i). Interestingly, *Hakai*^*SH2*^ and *Hakai*^*SH4*^ mutants phenocopy *Mettl3* adult defects in terms of the strong held-out wing (Fig. 3f–h) and 100% flightless phenotypes (Fig. 3i). Together, these data suggest that Hakai plays an important role in m^6^A-modification pathway in vivo.

### Hakai, Vir, Fl(2)d, and Flacc form a stable complex

**Fig. 4:**
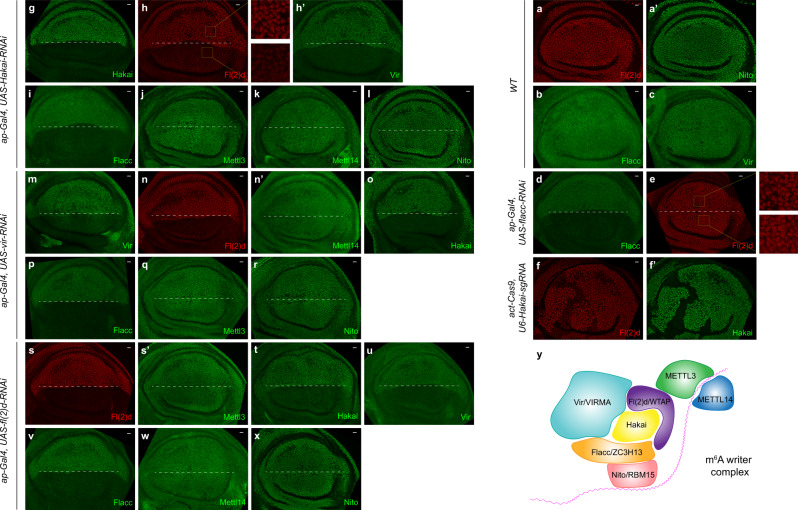
Hakai, Vir, Fl(2)d, and Flacc form a stable complex. In wild-type wing discs, Fl(2)d (**a**), Nito (**a’**), Flacc (**b**), and Vir (**c**) show ubiquitous nuclear staining patterns. **g**–**l** Expressing *Hakai* RNAi in the dorsal half of the disc (below the dashed line) using *ap-Gal4* resulted in a strong reduction of Fl(2)d (**h**, squared areas are magnified on the right), Vir (**h’**), and Flacc (**i**) levels, but not Mettl3 (**j**), Mettl14 (**k**), and Nito staining (**l**). **f**–**f’** Fl(2)d staining is similarly reduced in *Hakai* mutant clones generated by *actin-Cas9/U6-Hakai-sgRNA* (**f**), which are marked by the loss of Hakai staining (**f’**). **m**–**r** Expressing *vir* RNAi in the dorsal half of the disc (below the dashed line) using *ap-Gal4* resulted in a strong reduction of Fl(2)d (**n**), Hakai (**o**), and Flacc (**p**) levels, but not Mettl3 (**q**), Mettl14 (**n’**), and Nito staining (**r**). **s**–**x** Expressing *fl(2)d* RNAi in the dorsal half of the disc (below the dashed line) using *ap-Gal4* resulted in a strong reduction of Hakai (**t**), Vir (**u**), and Flacc (**v**) levels, but not Mettl3 (**s’**), Mettl14 (**w**), and Nito staining (**x**). **d**, **e** Expressing *Flacc* RNAi in the dorsal half of the disc (below the dashed line) using *ap-Gal4* led to more diffusive and less nuclear staining of Fl(2)d (**e**, squared areas are magnified on the right). The experiments in **a**–**x** were repeated at least twice independently with similar results, and each time around 30 wing discs for any genotype were examined. Scale bars: 10 μm. **y** A working model of the m^6^A writer complex comprised of seven core components.

We aim to investigate further the mechanisms of Hakai in m^6^A methylation. Since Hakai is a potential E3 ubiquitin ligase, we examined the protein distribution of other m^6^A writer factors in the absence of *Hakai*. For this purpose, we generated antibodies against Mettl3, Mettl14, and Vir, constituting a full toolkit for all seven *Drosophila* m^6^A writers. In wild-type wing discs, all seven m^6^A writer components appeared as ubiquitous nuclear proteins that colocalizes extensively with each other (Figs. 4a–c,  2c–c” and Supplementary Fig. 5a, c). Expression of *Hakai* RNAi in the dorsal half of the wing disc using *ap-Gal4* led to no effect on Mettl3, Mettl14, and Nito protein levels (Fig. 4j–l), but the strong reduction of Fl(2)d, Vir, and Flacc levels (Fig. 4h–i). In addition, we crossed *actin-Cas9* with *U6-Hakai-sgRNA* flies to generate random *Hakai* loss-of-function clones. These clones were marked by the loss of Hakai staining, and the Fl(2)d level was reduced in these clones as well (Fig. 4f–f’). Since the roles of several other m^6^A writer components are not fully understood, we extended our immunostaining assays to those genes. Interestingly, knocking down *vir* by RNAi resulted in no effect on Mettl3, Mettl14, and Nito protein levels (Fig. 4q, n’, r), but strong reduction of Fl(2)d, Hakai, and Flacc levels (Fig. 4n, o, p). Similarly, depletion of *fl(2)d* by RNAi did not change the protein levels of Mettl3, Mettl14, and Nito (Fig. 4s’, w, x), but strongly reduced Hakai, Vir, and Flacc levels (Fig. 4t–v). Together, these results suggest that Fl(2)d, Vir, Hakai, and Flacc form a stable complex, and disruption of either Fl(2)d, Vir, or Hakai leads to degradation of the whole complex, while Mettl3, Mettl14, and Nito are not part of this complex. In cell culture, ZC3H13 plays a role in anchoring the writer complex in the nucleus^35^. Consistent with this, we observed more diffusive and less nuclear staining of Fl(2)d upon *flacc* RNAi knockdown in the wing discs (Fig. 4d, e, compare insets in 4e with 4h). Based on our data, we proposed a working model for the m^6^A writer complex (Fig. 4y, see “Discussion”).

### Hakai does not mediate E-cadherin levels in wing discs

**Fig. 5:**
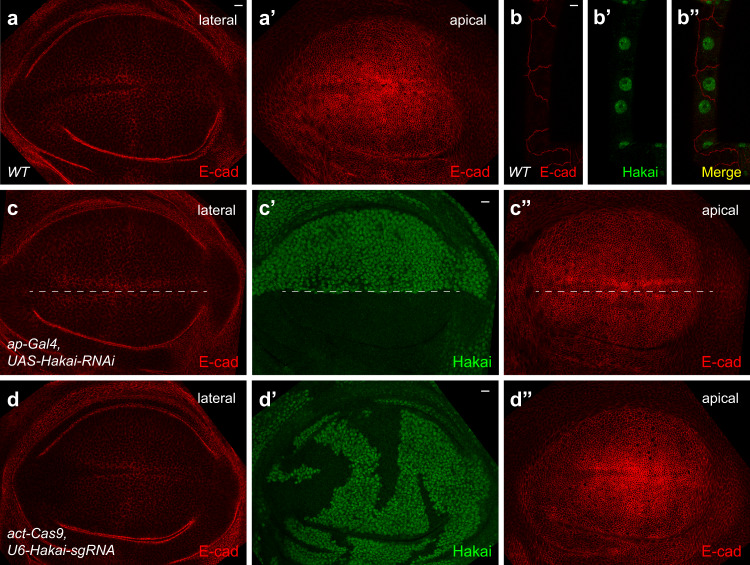
Hakai does not mediate E-cadherin levels in wing discs. **a**–**a’** Lateral (**a**) and apical (**a**’) section of E-cadherin staining in wild-type wing-disc epithelia. **b**–**b”** E-cadherin is mainly localized in the cell membrane (**b**), while Hakai is mostly in the nucleus (**b**’) in the large tracheal cells. **c**–**c”** Expression of *Hakai* RNAi in the dorsal half of the disc (below the dashed line) using *ap-Gal4* led to a strong reduction of Hakai (**c’**), but E-cadherin staining was not affected either laterally (**c**) or apically (**c”**). **d**–**d**” Lateral (**d**) and apical (**d**”) E-cadherin distribution is not changed in *Hakai* mutant clones marked by the absence of Hakai staining (**d’**), when crossing *actin-Cas9* with *U6-Hakai-sgRNA*. The experiments in **a-d”** were repeated at least twice independently with similar results, and each time around 30 wing discs for any genotype were examined. Scale bars: 10 μm.

Hakai was first demonstrated as an E-cadherin interaction protein and its role in E-cadherin endocytosis and down-regulation was extensively studied in cell cultures^50,51^. However, a previous study in *Drosophila* failed to observe a major role for Hakai in E-cadherin regulation^52^. Thus, we addressed this question using our genetic toolset. In wild-type wing-disc epithelia, E-cadherin showed a membrane distribution and accumulates in the adherens junction (Fig. 5a, a’). First, we used *ap-Gal4* to drive the expression of *Hakai* RNAi in the dorsal half of the wing disc. Although Hakai level was effectively knocked down (Fig. 5c’), E-cadherin level did not change in either the apical or the lateral section (Fig. 5c, c”). Second, in *Hakai* mutant clones generated by crossing *actin-Cas9* with *U6-Hakai-sgRNA*, E-cadherin level was not affected either (Fig. 5d–d”). Finally, we examined the localization of E-cadherin and Hakai in detail using large tracheal cells. As shown in Fig. 5b–b”, most E-cadherin staining was on the cell membrane, while most Hakai staining was in the nucleus, and we did not observe co-localization between these two proteins. In conclusion, Hakai is not important for E-cadherin levels in *Drosophila*, and its major function likely happens in the nucleus.

### The m^6^A modifications are deposited onto the *Sxl* mRNA in a sex-specific fashion

**Fig. 6:**
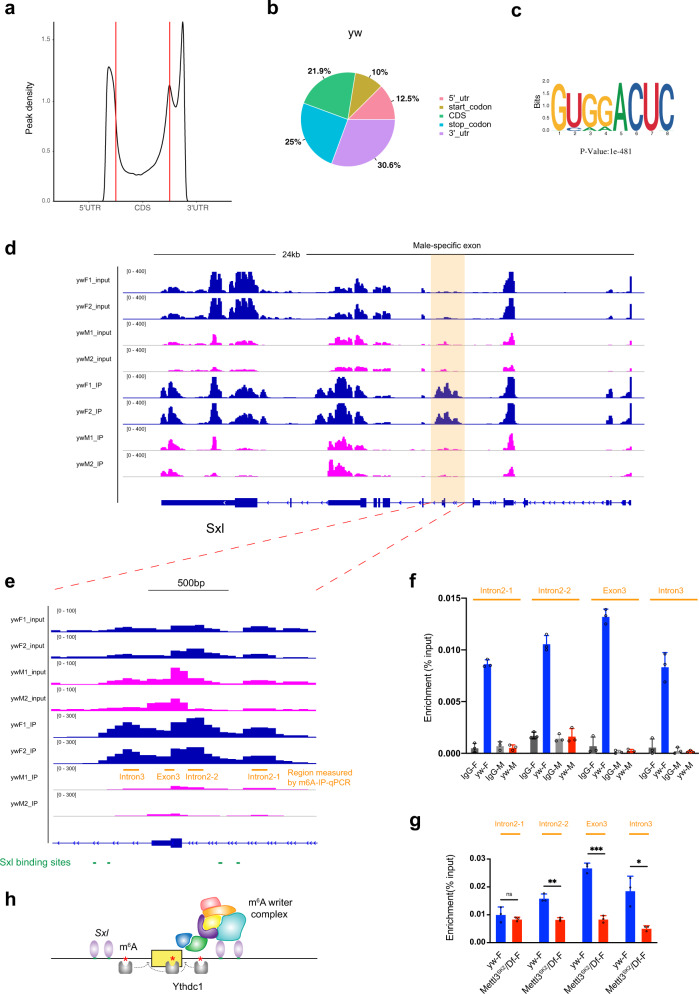
Female-specific deposition of m^6^A on the *Sxl* mRNA. **a** MeRIP-seq shows that the normalized density of m^6^A peaks across 5′UTR, CDS, and 3′UTR of mRNA in *yw* male adult flies. **b** Pie charts depicting m^6^A peak distribution in different transcript segments in *yw* flies. **c** Sequence motif identified from m^6^A peaks in *yw* flies by HOMER program. **d** Integrative Genomics Viewer (IGV) tracks displaying MeRIP-seq (lower panels, IP) and RNA-seq (upper panels, input) reads along *Sxl* locus in *yw* male (ywM) and female flies (ywF). Two replicates are shown. The region around male-specific exon3 is shaded and enlarged in (**e**) highlighting 3–4 m^6^A peaks on and around exon3 only in female flies. Adjacent Sxl-binding sites are also shown. **f** m^6^A-IP-qPCR showing enrichments over *Sxl* mRNA in *yw* male or female flies IPed with m^6^A or IgG antibody. Regions measured are indicated in (**e**). **g** m^6^A-IP-qPCR showing enrichments over *Sxl* mRNA in *yw* or *Mettl3*^*SK2*^*/Df* female flies IPed with m^6^A antibody. Data are presented as mean ± SD from three biological replicates. *P < 0.05; **P < 0.01; ***P < 0.001; ****P < 0.0001; ns not significant, two-sided unpaired *t* test. In **g**, *P* = 0.3679, 0.0012, 0.0001, 0.0114. Source data are provided as a Source Data file. **h** A working model on how the m^6^A modifications cooperate with Sxl to regulate its mRNA splicing.

Previously, only two studies were reported to map global m^6^A methylation pattern in *Drosophila*, one being MeRIP-seq in S2R + cells and the other being miCLIP in embryos^33,47^. To obtain a high-stringent m^6^A methylome in adult flies, we performed methylated RNA immunoprecipitation sequencing (MeRIP-seq) in *yw* male and female flies. We included two replicates for each MeRIP-seq and the mapped reads were enriched around TSS and TES in IP samples compared to inputs (Supplementary Fig. 6). A peak detection algorithm was used to identify m^6^A peaks (*P* < 0.05, Supplementary Data 1), which were enriched in the 3′UTR and close to the stop codon, and to a lesser extend enriched in the 5′UTR and around the start codon (Fig. 6a, b). De novo motif analysis using HOMER identified the consensus sequence RRACH (Fig. 6c), consistent with those in the mammalian system^8,9^.

We then zoomed in on the *Sxl* locus, especially around the male-specific exon3, for potential m^6^A sites (Fig. 6d). Although the mRNAs used for MeRIP-seq were through polyA selection, we were able to detect 3–4 m^6^A peaks in and around exon3 (Fig. 6e). Strikingly, these m^6^A peaks only exist in female flies, but not males (Fig. 6e, compare ywF_IP with ywM_IP). We next validated the MeRIP-seq results with independent m^6^A-immunoprecipitation (IP)-qPCR. Four regions on *Sxl* mRNA, intron2–1, intron2–2, exon3, and intron3, were measured by qRT-PCR (Fig. 6e), and substantial enrichment was observed only in female mRNA IPed with m^6^A-specific antibody, but not in female mRNA IPed with control IgG, nor in male mRNA IPed with either m^6^A or IgG antibody (Fig. 6f). These results demonstrated that the m^6^A modifications are deposited in a sex-specific manner, which has not been shown in *Drosophila* or any other species before. We further investigated whether m^6^A modifications around *Sxl* exon3 are dependent on the m^6^A writer. In *Mettl3* mutant females, the enrichments on intron2-2, exon3, and intron3, but not intron2-1, were significantly reduced, probably implying the importance of the former three m^6^A sites (Fig. 6g).

How can these m^6^A modifications be installed only in females? In fact, Sxl protein itself is the master sex determination factor that is only expressed in females. Sxl binds to polyU sites located in *Sxl* intron2 and intron3^61,62^ and interestingly our mapped m^6^A peaks were close to those Sxl-binding sites (Fig. 6e). In addition, it was known that Sxl physically interacts with four m^6^A writer components, Fl(2)d, Vir, Nito, and Flacc^36,41,42,45,46^. Therefore, based on our data, we developed a model to explain how the m^6^A modifications cooperate with Sxl protein to regulate its mRNA splicing (Fig. 6h). Sxl in females recruits the m^6^A writer complex that in turn methylates m^6^A sites located in exon3 and nearby introns. Since these sites are quite close to exon/intron junction regions, m^6^A readers may bind to these sites and interfere with the splicing machinery, forcing the exon3 to be skipped in females (Fig. 6h).

### Effective m^6^A modification occurs in 5′UTR and around start codon in *Drosophila*

**Fig. 7:**
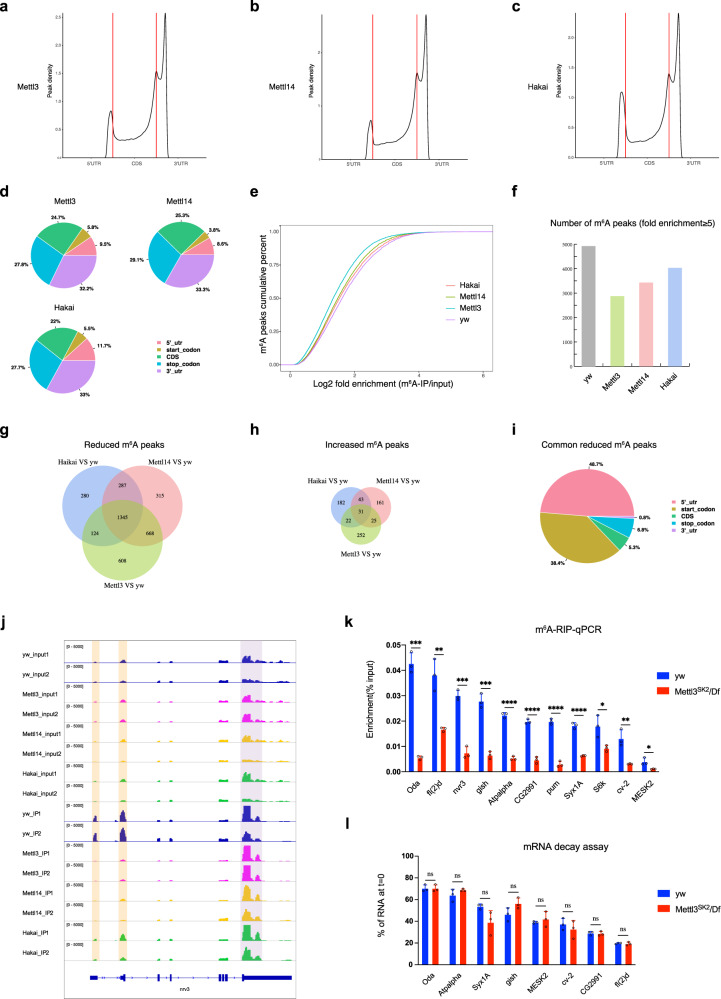
Effective m^6^A modification in *Drosophila* is distributed in 5′UTRs. **a**–**c** MeRIP-seq shows the normalized density of m^6^A peaks across 5′UTR, CDS, and 3′UTR of mRNA in *Mettl3*, *Mettl14*, and *Hakai* mutant male adult flies. **d** Pie charts depicting m^6^A peak distribution in different transcript segments in *Mettl3*, *Mettl14*, and *Hakai* flies. **e** Cumulative percent distribution of log2 fold enrichment (m^6^A-IP/input) in *yw*, *Mettl3*, *Mettl14*, and *Hakai* flies. **f** When filtered with fold enrichment ≥5, the number of m^6^A peaks dropped in *Mettl3, Mettl14*, or *Hakai* mutant compared with *yw*. **g** Venn diagram showing the overlap of reduced m^6^A peaks (*P* < 0.05 and fold change ≤0.5) between *Mettl3*, *Mettl14*, and *Hakai* flies versus *yw* control. **h** Venn diagram showing the overlap of increased m^6^A peaks (*P* < 0.05 and fold change ≥2) between *Mettl3*, *Mettl14*, and *Hakai* flies versus *yw* control. **i** Pie chart depicting the distribution of 1345 common reduced m^6^A peaks in different transcript segments. **j** IGV tracks displaying MeRIP-seq (lower panels, IP) and RNA-seq (upper panels, input) reads along *nrv3* mRNA in *yw*, *Mettl3*, *Mettl14*, and *Hakai* male flies. Note the reduced peaks in the 5′UTR (shaded in yellow) and peaks in the 3′UTR (shaded in purple) are not changed. **k** m^6^A-IP-qPCR validation for selected mRNAs in *yw* or *Mettl3*^*SK2*^*/Df* male flies. **l** The m^6^A-containing mRNAs were measured by qPCR in *yw* or *Mettl3*^*SK2*^*/Df* imaginal discs, at time = 0 or after four hours actinomycin D treatment to block transcription. **k**, **l** Data are presented as mean ± SD from three biological replicates. *P < 0.05; **P < 0.01; ***P < 0.001; ****P < 0.0001; ns not significant, two-sided unpaired *t* test. In **k**, *P* = 0.0001, 0.0043, 0.0003, 0.0003, <0.0001, <0.0001, <0.0001, <0.0001, =0.0273, 0.0073, 0.0276 for *Oda*, *fl(2)d*, *nvr3*, *gish*, *Atpalpha*, *CG2991*, *pum*, *Syx1A*, *S6k*, *cv-2*, *MESK2*. In **l**, *P* > 0.9999, =0.2282, 0.0832, 0.0939, 0.5032, 0.455, 0.8416, 0.7415 for *Oda*, *Atpalpha*, *Syx1A*, *gish*, *MESK2*, *cv-2*, *CG2991*, *fl(2)d*. Source data are provided as a Source Data file.

Since previous m^6^A-Seq data in *Drosophila* were generated only in wild-type cells or tissues^33,47^, we extended our MeRIP-seq to *Mettl3*, *Mettl14*, and *Hakai* mutants in order to find out the effective m^6^A modifications that depend on the m^6^A writer (Supplementary Fig. 6 and Supplementary Data 1). We used previously reported alleles *Mettl3*^*SK2*^ and *Mettl14*^*SK1*^ for our experiments^47^, since they are strong or null alleles as evidenced by the complete loss of Mettl3 and Mettl14 antibody staining in these mutant discs, respectively (Supplementary Fig. 5). Peak density plots indicated that m^6^A peaks were primarily enriched in the 3′UTR and near the stop codon, and enrichment in the 5′UTR and around the start codon seems to be reduced in these mutants compared to *yw* (Fig. 7a–d, compare to 6a). The cumulative distribution plot demonstrated that there is less fold enrichment (m^6^A-IP/input) in m^6^A mutants than *yw*, implying less methylation levels (Fig. 7e). Indeed, when we filtered m^6^A peaks with higher stringency (fold enrichment ≥5), the number of m^6^A peaks dropped >40% in *Mettl3* mutant compared with *yw* (Fig. 7f).

More importantly, we focused on those peaks changed upon m^6^A writer depletion. Interestingly, many more m^6^A peaks were reduced (*Mettl3*, 2745; *Mettl14*, 2615; *Hakai*, 2036; *P* < 0.05 and fold change ≤0.5) than increased (*Mettl3*, 330; *Mettl14*, 260; *Hakai*, 278, *P* < 0.05 and fold change ≥2) in these mutants, validating their role as m^6^A writers (Fig. 7g, h and Supplementary Data 2). Only in reduced m^6^A peaks, a significant overlap between the three mutants was found (1345 common peaks), suggesting that Hakai plays a similar role to Mettl3 and Mettl14 in m^6^A methylation. The common reduced peaks in three writer mutants likely represent high-confidence m^6^A modification sites in *Drosophila*. We focused on common reduced m^6^A peaks for further analysis. Surprisingly, around 90% of these peaks were located in the 5′UTR and close to the start codon, and only a small portion of them occurred in the 3′UTR (Fig. 7i). A few examples of loss of m^6^A peaks in the three mutants were shown in Fig. 7j and Supplementary Fig. 7, which almost always happened in the 5′UTR, while m^6^A peaks in the 3′UTR were generally not changed. We then used m^6^A-IP-qPCR to validate the reduction of m^6^A peaks in the 5′UTR region for 11 genes and found a significant reduction of m^6^A signal in *Mettl3* mutant versus *yw* (Fig. 7k). These results indicate that although most m^6^A sites map to 3′UTRs in *Drosophila*, the peaks responding to the loss of m^6^A writers, and thus the effective ones, are distributed in 5′UTRs and this is different from the mammalian system. The majority of the peaks in 3′UTRs may be mediated by another methyltransferase or come from a non-specific background.

Next, we analyzed gene expression regulated by m^6^A writer components by RNA-seq (Supplementary Data 3). In *Mettl3*, *Mettl14*, and *Hakai* mutant adult flies, 987, 954, and 886 genes were differentially expressed (*P* < 0.05 and fold change ≥2 or ≤0.5), respectively (Supplementary Fig. 8a and Supplementary Data 4). These genes substantially overlapped with each other (Supplementary Fig. 8b) and common differentially expressed genes generally changed in the same pattern in the three mutants (Supplementary Fig. 8c), arguing that they act in the same pathway. Interestingly, differentially expressed genes were strongly enriched for immune response genes in the GO analysis and for metabolic pathways in the KEGG analysis (Supplementary Fig. 8d, e). By exhibiting MeRIP-seq together with RNA-seq data, we observed the reduction of m^6^A peaks in *Mettl3*, *Mettl14*, or *Hakai* mutant, and no obvious trend for change of mRNA expression correlated with differential m^6^A peaks (Supplementary Fig. 8f–h and Supplementary Data 5). We further divided the genes into m^6^A targets or nontargets, and the cumulative plot shows that there was no obvious difference between m^6^A targets and nontargets in *Hakai* or *Mettl3* mutant flies (Supplementary Fig. 8i, j), while there was a slight positive effect of m^6^A on mRNA levels in *Mettl14* mutant (Supplementary Fig. 8k), consistent with a previous report^33^. Moreover, we performed RNA decay assay for validated m^6^A-containing transcripts. Four hours after transcription inhibition by actinomycin D, we did not observe the significant difference in mRNA levels for these genes between *Mettl3* and *yw* imaginal discs (Fig. 7l). These findings are consistent with the notion that effective m^6^A modifications are located in 5′UTRs in *Drosophila*, and thus do not mediate mRNA degradation as in the mammalian system.

Finally, we performed a similar analysis for splicing changes. In *Mettl3*, *Mettl14*, and *Hakai* mutant flies, 445, 364, and 340 genes were differentially spliced (FDR < 0.05, IncLevelDifference ≥0.2 or ≤ −0.2), respectively, and they overlapped substantially with each other (Supplementary Fig. 9a, c and Supplementary Data 6–8). The differential alternative spliced events occurred in all splicing categories (Supplementary Fig. 9b) and were functionally enriched for mRNA splicing and signaling transduction, etc (Supplementary Fig. 9d, e). We then examined the alternatively spliced transcripts of *Dsp1*, *CG8929*, *Aldh-III*, and *fl(2)d*, which were regulated by the m^6^A pathway (Fig. 3c). In all four cases, we found heavily methylated peaks surrounding the splicing junctions that depended on Mettl3, suggesting that these modifications may regulate splicing directly (Supplementary Fig. 9f–i). Interestingly, it seemed that the role of m^6^A modification is to repress splicing in these events. Even in the case of *Sxl*, the m^6^A modification plays a role to inhibit splicing as well, whether this is a general mechanism needs to be determined in the future.

## Discussion

m^6^A modification has been known for more than 40 years^6^ but has recently gained great attention due to the emergence of technologies to map m^6^A methylome^8,9,63^, as well as the identification of the writers, readers, and erasers in this pathway^4,14–16^. Since the initial purification of the key methyltransferase Mettl3^21^, other components of the writer complex were gradually identified through biochemical experiments and genetic screens. We now know that m^6^A writer complex is comprised of multiple components including Mettl3, Mettl14, WTAP, VIRMA, RBM15/15B, ZC3H13. Hakai was first indicated as a WTAP interaction protein^23^ and was shown later to be required for full m^6^A methylation in *Arabidopsis*^37^; however, its role in the m^6^A pathway in animals has not been studied. Here, we show that Hakai interacts with other m^6^A writer subunits, and *Hakai* mutants exhibit characteristic m^6^A pathway phenotypes, such as lowered m^6^A levels in mRNA, aberrant alternative splicing of *Sxl* and other genes, held-out wings, and flightless flies, as well as reduced m^6^A peaks shared with *Mettl3* and *Mettl14* mutants in MeRIP-seq. Altogether, these data unambiguously argue that Hakai is the seventh, and likely last core component of the conserved m^6^A writer complex.

Each component in the m^6^A writer complex plays a role in mRNA methylation but their exact roles are not well understood^64^. Our systematic analysis of several m^6^A writer subunits has provided insights into the mechanism of this important complex. We found that Fl(2)d, Vir, Hakai, and Flacc form a stable complex, and knocking down either of Fl(2)d, Vir, or Hakai led to the degradation of the other three components. Mettl3, Mettl14, and Nito were not affected by the disruption of Fl(2)d, Vir or Hakai, suggesting that they have separate functions. Knocking down Flacc resulted in less nuclear staining of Fl(2)d, consistent with a role in nuclear localization of the writer complex^35^. Based on these results, we proposed a model for the m^6^A methyltransferase complex (Fig. 4y). Mettl3 and Mettl14 form a stable heterodimer to catalyze the addition of the methyl group to mRNA. Nito/RBM15 contains three RRM domains and binds to positions adjacent to m^6^A sites, thus may provide target specificity for the m^6^A writer complex^32^. Fl(2)–Vir–Hakai–Flacc form a platform to connect different components and may integrate environmental and cellular signals to regulate m^6^A methylation.

Hakai is a potential E3 ubiquitin ligase with an intact C3HC4 RING domain and a C2H2 domain. Its absence led to the degradation, rather than the accumulation of other m^6^A writer subunits, indicating that it may not act as an E3 ubiquitin ligase in this complex. Hakai was initially identified as an E-cadherin binding protein to downgrade its levels^50^, and the role of Hakai in cell proliferation and tumor progression was extensively studied in cell culture^51^. However, our in vivo analysis using various genetic tools did not find a role of Hakai in E-cadherin regulation. In addition, Hakai appeared as a ubiquitous nuclear protein showing little co-localization with E-cadherin in the membrane. Consistently, Hakai was shown to interact with PTB-associated splicing factor (PSF), a nuclear protein, and to affect its RNA-binding ability^65^. Thus, the role of Hakai in E-cadherin regulation needs to be further investigated using the knockout mouse model and whether Hakai has other substrates for its E3 ligase activity also needs to be determined.

Recent emerging studies suggest that m^6^A is involved in numerous developmental processes and human diseases^16,66^, mainly by regulating mRNA stability, translation, or splicing. Pioneer work from three labs has established the framework for the m^6^A pathway in *Drosophila*^33,47,48^. However, only published *Drosophila* m^6^A methylome was performed in S2R + cells or embryos^33,47^ and was not done against writer mutants. Other than *Sxl*, few m^6^A target loci have been firmly mapped. By performing MeRIP-seq in wild-type adult flies as well as *Mettl3*, *Mettl14*, and *Hakai* mutants, we demonstrated that although most m^6^A peaks are distributed in 3′UTRs, the functional peaks responding to the loss of m^6^A writers are mainly located in 5′UTRs. This finding indicates a major difference between *Drosophila* and mammalian m^6^A methylome, which mainly occurs in 3′UTRs, and is in agreement with a recently published manuscript using miCLIP^67^. Interestingly, our LC-MS data show that the overall level of m^6^A modification in *Drosophila* only accounted for 10–20% of that in mammalian cells. *Mettl3* or *Mettl14* mutants are embryonic lethal in mice while they develop into adults in flies. It is possible that the m^6^A pathway acquires additional functions during evolution.

m^6^A modification in 3′UTRs usually causes mRNA instability and m^6^A in 5′UTRs is linked to translation enhancement^1,4^. In agreement with the view that functional m^6^A peaks are located in 5′UTRs in *Drosophila*, we did not observe an increase in mRNA half-life of m^6^A targets in *Mettl3* mutants compared to wild-type. These results imply that the major role of m^6^A modification in *Drosophila* is not on mRNA degradation, but possibly on translation upregulation, which can be tested by combining ribosome profiling^68^ and functional analysis of a single transcript in the future. Our data by combining MeRIP-seq and splicing analysis shed light on how the m^6^A modification contributes to splicing regulation. In all five cases, we analyzed, four (*Dsp1*, *CG8929*, *fl(2)d*, *Aldh-III*) in 5′UTRs and one (*Sxl*) in exon/intron, reduction of m^6^A modification was correlated with enhanced splicing, arguing that the normal role of these modifications might be to repress splicing events nearby.

Last but probably the most interesting finding from our work is to demonstrate the female-specific m^6^A modification around *Sxl* exon3. *Sxl* is a textbook paradigm to study alternative splicing and has been intensively investigated for more than thirty years^69^. Sxl protein binds to its own mRNA to control the alternative splicing, but its binding sites are located >200 nucleotides downstream or upstream of the male exon, meaning other regulators should be involved. Recently, the m^6^A modification pathway was shown to modulate *Sxl* alternative splicing, but the detailed mechanism has not been resolved^33,47,48^. Our MeRIP-seq data revealed that several m^6^A peaks were deposited only in females on and around *Sxl* exon3, and they were in the vicinity of Sxl-binding sites (Fig. 6e). We further validated this finding by independent m^6^A-IP-qPCR and showed that these modifications were reduced in *Mettl3* mutant females. This unexpected finding suggests a model that one main function of Sxl may be to recruit the m^6^A writer complex that methylates nearby m^6^A sites. The m^6^A reader Ythdc1 in turn binds to these sites and might interact with the splicing machinery to repress splicing (Fig. 6h). Future experiments, such as interactions between Sxl and Mettl3/Mettl14, interactions between Ythdc1 and general splicing factors, mapping of the exact m^6^A methylation site in *Sxl* at the single nucleotide level, comparison of transcriptome-wide binding sites of Sxl with m^6^A modification sites, will be required to firmly prove our model.

## Methods

### Fly strains and genetics

Flies were grown on standard cornmeal food and experiments were performed at 25 °C. The following stocks were used: *w*^*1118*^ (used as wild-type, WT), *yw*, *Mettl3*^*SK2*^, *Mettl14*^*SK1*^ ^47^, *Df(3R)Exel6197* (*Mettl3* deficiency, Bloomington 7676), *ap-Gal4*, *Hakai* shRNA (VDRC330548), *vir shRNA* (HMC03908, Bloomington 55694), *fl(2)d shRNA* (HMC03833, Bloomington 55674), *flacc shRNA* (VDRC 35212GD), *actin-Cas9* (Bloomington 54590)^55^, *nanos-Cas9* (Bloomington 78782)^57^. To generate *U6-Hakai-sgRNA*, target sequence “ACGTCCGCGCCCGCGAGCCC” was picked by DRSC Find CRISPRs^57^ and cloned into pCFD3 vector^55^ (Addgene 49410). The construct was inserted at attP40 site by standard PhiC31-mediated transformation to make transgenic flies in UniHuaii. *U6-Hakai-sgRNA* was crossed with *nanos-Cas9* flies to generate a series of indel mutations, and *Hakai*^*SH2*^ and *Hakai*^*SH4*^ were chosen for further analysis. Two additional Hakai shRNAs were made based on the pNP vector by Tsinghua Fly Center^56^. TH14422.S with target sequence “GAGCTCGACAAGGACGGCGAA” was inserted at attP2 site and TH14423.S with target sequence “CGGCCGCATGATACCCTGCAA” was inserted at attP40 site. The three *Hakai* shRNAs show similar knockdown efficiency as examined by Hakai antibody staining. VDRC330548 was used in Figs. 4g, j, k, 5c–c”, TH14423.S was used in Fig. 4h–h’, 4i, and TH14422.S was used in Fig. 4l. To test adult flight ability, cohorts of ten male flies were tapped down into a Petri dish, and the number of flies that flew away within 2 min was recorded.

### Immunostaining

Wing discs from 3rd instar larvae were dissected in PBS and fixed in 4% formaldehyde (Sigma) in PBST (PBS + 0.1% Triton X-100) for 15 min. After blocking in 1% normal donkey serum (Jackson Immuno) in PBST for 1 h, the samples were incubated with the primary antibody in the same solution at 4 °C overnight. After three washes in PBST, samples were incubated with the secondary antibody for 2 h at room temperature, washed in PBST three times, and subsequently mounted in Antifade Mounting Medium (Beyotime). All images were taken on a Zeiss LSM 880 microscope.

The following antibodies were used: mouse anti-Fl(2)d (1:10) (9G2, DSHB), rat anti-Ecad (1:5) (DCAD2, DSHB), mouse anti-Sxl (1:10) (M18, DSHB), rabbit anti-Flacc (1:200)^36^, rabbit anti-Hakai (1:200), rabbit anti-Vir (1:200), rabbit anti-Mettl3 (1:200), rabbit anti-Mettl14 (1:200); Alexa 488-conjugated anti-mouse and anti-rabbit IgG (1:1000) (Thermo Fisher, A21202, A21206), Cy3-conjugated anti-mouse, and anti-rabbit IgG (1:400) (Jackson Immuno, 715-165-150, 711-165-152). Hakai antibody was generated in rabbits against a recombinant protein containing amino acids 1–120. Vir antibody was generated in rabbits against a recombinant protein containing amino acids 750–1005. Mettl3 antibody was generated in rabbits against a recombinant protein containing amino acids 110–300. Mettl14 antibody was generated in rabbits against a recombinant protein containing amino acids 1–205. All four antibodies were generated and affinity-purified at ABclonal.

### Molecular cloning and co-immunoprecipitation

To generate the GFP-, mRFP-, or HA-tagged plasmids, full-length cDNAs for Hakai (the long and the short isoform amplified from a cDNA library), Flacc (GH14795), Nito (GH11110), Fl(2)d (LD21616), METTL3 (AT20169), and METTL14 (LD06016) were cloned into the pENTR vector (Invitrogen), and transferred into the *Drosophila* Gateway vector pAGW, pARW, and pAHW. GFP was cloned into pAWM as a control.

*Drosophila* S2 cells were maintained in Schneider’s medium (Gibco) supplemented with 10% FBS (Gibco) at 25 °C. In all, 4 μg of total DNA was transfected into S2 cells in a 100-mm dish with Effectene (QIAGEN). After 48 h, cells were lysed in IP lysis buffer (Beyotime, 50 mM Tris (pH 7.4), 150 mM NaCl, 1% NP-40, 1× protease inhibitor) on ice for 30 min, and cleared at 20,000×*g* for 10 min at 4 °C. Supernatants were incubated with anti-GFP nanobody agarose beads (Allele Biotechnology) for 2 h at 4 °C. The beads were washed three to four times with 1 ml lysis buffer and resuspended in 2× SDS sample buffer. Eluted proteins were detected by western blotting using rabbit anti-GFP (1:1000, A6455, Molecular Probes), rat anti-HA (1:1000, 3F10, Roche) or mouse anti-Fl(2)d (1:100, 9G2, DSHB) primary antibodies, and HRP-conjugated secondary antibodies (1:3000, Santa Cruz Biotech). Uncropped western blots can be found in the Source Data file. For co-localization, S2 cells were grown on Lab-Tek chamber slides and imaged in live conditions 2 days after transfection. HeLa cells were cultured at 37 °C with 5% CO_2_ in Dulbecco’s modified eagle’s medium (TransGen) supplemented with 10% FBS (Gibco).

### Analyzing m^6^A levels by LC-MS

Total RNAs were extracted from adult flies or cultured cells using TRIzol (Invitrogen), and then subjected to two rounds of poly(A) selection using the GenElute mRNA Miniprep kit (Sigma). Before LC-MS analysis, all RNA samples were hydrolyzed enzymatically to ribonucleosides. Briefly, 600 ng RNA of each sample was digested by nuclease P1 (Sigma) and snake venom phosphodiesterase (Sigma) at 37 °C for 2 h, and followed by digestion with fast alkaline phosphatase (Thermo Fisher) for another 1 h. Then, the digested samples were used for the following LC-MS analysis.

Quantitative LC-MS analyses of m^6^A and adenosine were achieved using a Waters UPLC coupled to Thermo Q Exactive mass spectrometer in positive ion mode using dynamic multiple reaction monitoring. The ribonucleosides in the hydrolyzed RNA samples were resolved on an Acquity UPLC HSS T3 column (1.8 µm particle size, 100 Å pore size, 2.1 × 100 mm, 30 °C) at 300 µl min^−1^ using a solvent system of 0.1% formic acid in H_2_O (A) and acetonitrile (B). The elution profile was 2% B for 2 min, 2–11% B over 4 min, then to 11–80% B over 4 min, followed by a column washing at 80% B and column equilibration. The quantification of a ribonucleoside can be achieved using *m/z* of the parent ribonucleoside ion and m/z of the deglycosylated ion product. Nucleosides were quantified based on the transition of the parent ribonucleoside to the deglycosylated base ion: *m/z* 268.1–136.1 for A, *m/z* 282.1–150.1 for m^6^A, *m/z* 282.1–150.1 for m^1^A, *m/z* 244.1–112 for C, *m/z* 258.1–126 for m^5^C, and *m/z* 286.1–154 for ac^4^C. Absolute quantities of each ribonucleoside were determined using an external calibration curve prepared with A (Sigma), m^6^A (Selleck), m^1^A (J&K), C (Sigma), m^5^C (Sigma), and ac^4^C (J&K) standards.

### MeRIP-seq

MeRIP-seq was performed following previous protocols^70,71^. The total RNA was isolated from *yw* (male and female), *Mettl3*^*SK2*^*/Df* (male), *Mettl14*^*SK1*^ (male), and *Hakai*^*SH4*^ (male) adult fly about 1–2 days old using TRIzol (Invitrogen). The RNA amount of each sample was quantified using NanoDrop ND-1000 and the RNA integrity was assessed by Bioanalyzer 2100 (Agilent) with RIN number >7.0, and confirmed by electrophoresis with denaturing agarose gel. Poly(A) RNA was purified from 50 μg of the total RNA using Dynabeads Oligo (dT)25 (Thermo Fisher) by two rounds of purification and then was fragmented into small pieces using Magnesium RNA Fragmentation Module (NEB) under 86 °C for 7 min. The cleaved RNA fragments were incubated for 2 h at 4 °C with Dynabeads (Dynabeads Antibody Coupling Kit, Thermo Fisher) coupled with m^6^A-specific antibody (202003, Synaptic Systems) in IP buffer (50 mM Tris-HCl, 750 mM NaCl, and 0.5% Igepal CA-630). Then the IP RNA fragments and untreated input control fragments are converted to the final cDNA library in accordance with a strand-specific library preparation by the dUTP method. The average insert size for the final cDNA library was 200 ± 50 bp. At last, we performed the 2 × 150 bp paired-end sequencing (PE150) on an Illumina Novaseq 6000 (LC-Bio Technology) following the vendor’s recommended protocol.

### Bioinformatic analysis

fastp (v0.19.4) software was used to remove the reads that contained adaptor contamination, low-quality bases, and undetermined bases with default parameters. Then sequence quality of IP and Input samples were also verified using fastp. We used HISAT2 (v2.0.4)^72^ to map reads to the genome of *Drosophila_melanogaster* (Version: v96) with default parameters. Mapped reads of IP and input libraries were provided for R package exomePeak (v1.9.1)^73^, which identifies m^6^A peaks with bed or bigwig format that can be adapted for visualization on the IGV software. HOMER (v4.1)^74^ was used for de novo and known motif finding followed by localization of the motif with respect to peak summit. Called peaks were annotated by the intersection with gene architecture using R package ChIPseeker (v1.18.0)^75^. A common peak was picked if the peaks from different groups overlap in the genome >50% of the smallest of the peaks. StringTie (v1.3.4)^76^ was used to perform expression levels for all mRNAs from input libraries by calculating FPKM. The differentially expressed mRNAs were selected with log2 (fold change) ≥1 or log2 (fold change) ≤−1 and *P* value <0.05 by R package edgeR (v4.1)^77^. rMATS (v4.0.1) was used for differential splicing analysis with a filter for FDR < 0.05 and IncLevelDifference ≥0.2 or ≤ −0.2^78^.

### RT-PCR

Total RNAs were extracted using TRIzol (Invitrogen) and cDNAs were generated from 0.5 μg of RNA using Hifair II 1st Strand cDNA Synthesis Kit with gDNA digester plus (Yeason). 2xHieff PCR Master Mix (Yeasen) was used for regular PCR, and Hieff qPCR SYBR Green Master Mix (Yeasen) was used for qPCR. All primers used are listed in Supplementary Data 9. *Sxl* primers used in Fig. 3b are described in ref. ^59^ and *Dsp1*, *CG8929*, *Aldh-III* and *fl(2)d* primers used in Fig. 3c are described in refs. ^33,34^. Statistical analysis was performed using GraphPad Prism (v9.0.0).

### m^6^A-IP-qPCR

m^6^A-IP-qPCR was performed following a protocol with minor modifications^70^. Total RNAs were extracted using TRIzol (Invitrogen) and purified through GenElute mRNA Miniprep kit (Sigma). In total, 5 µg of purified mRNAs were fragmented into ~200 300 nt fragments by incubation in RNA fragmentation reagent (Thermo Fisher) at 94 °C for 30 s and then stopped with stop solution. Ten percent of the fragmented mRNAs were kept as input. The remaining mRNAs were incubated with 6.25 µg m^6^A-specific antibody (202003, Synaptic Systems) or rabbit IgG (Beyotime) in IP buffer (50 mM Tris-HCl (pH 7.4), 750 mM NaCl, 0.5% Igepal CA-630, 0.4 U/µl RNasin (Promega)) for 2 h at 4 °C. Then the mixture was incubated with 25 µl Dynabeads Protein A (Thermo Fisher) for another 2 h at 4 °C. After extensive washing, the bound mRNAs were eluted with 6.7 mM N6-methyladenosine (Sigma) in IP buffer and then recovered with ethanol precipitation. The immunoprecipitated mRNAs and input mRNAs were processed as in RT-PCR.

### mRNA decay assay

To assay for mRNA stability, *yw* or *Mettl3* mutant L3 larval imaginal discs were dissected and treated with 20 µg/ml Actinomycin D (Sigma) in Schneider’s medium (Gibco) for 4 h at 25 °C. Total RNA was extracted at *T* = 0 h and *T* = 4 h and processed as in RT-PCR.

### Reporting summary

Further information on research design is available in the Nature Research Reporting Summary linked to this article.

## Supplementary information

Supplementary information

Peer Review File

Supplementary Data 1

Supplementary Data 2

Supplementary Data 3

Supplementary Data 4

Supplementary Data 5

Supplementary Data 6

Supplementary Data 7

Supplementary Data 8

Supplementary Data 9

Description of additional supplementary files

Reporting Summary

## Data Availability

High-throughput m^6^A-seq and RNA-Seq data are deposited into the Gene Expression Omnibus with accession number GSE155662. The data supporting the findings of this study are available from the corresponding authors upon reasonable request. Source data are provided with this paper.

## Source data

Source Data

## References

[1] Zhao BS, Roundtree IA, He C (2017). Post-transcriptional gene regulation by mRNA modifications. Nat. Rev. Mol. Cell Biol..

[2] Gilbert WV, Bell TA, Schaening C (2016). Messenger RNA modifications: form, distribution, and function. Science.

[3] Boccaletto P (2018). MODOMICS: a database of RNA modification pathways. 2017 update. Nucleic Acids Res..

[4] Zaccara S, Ries RJ, Jaffrey SR (2019). Reading, writing and erasing mRNA methylation. Nat. Rev. Mol. Cell Biol..

[5] Roundtree IA, Evans ME, Pan T, He C (2017). Dynamic RNA modifications in gene expression regulation. Cell.

[6] Desrosiers R, Friderici K, Rottman F (1974). Identification of methylated nucleosides in messenger RNA from Novikoff hepatoma cells. Proc. Natl Acad. Sci. USA.

[7] Perry R, Kelley DE (1974). Existence of methylated messenger RNA in mouse L cells. Cell.

[8] Meyer KD (2012). Comprehensive analysis of mRNA methylation reveals enrichment in 3’ UTRs and near stop codons. Cell.

[9] Dominissini D (2012). Topology of the human and mouse m6A RNA methylomes revealed by m6A-seq. Nature.

[10] Schwartz S (2013). High-resolution mapping reveals a conserved, widespread, dynamic mRNA methylation program in yeast meiosis. Cell.

[11] Luo GZ (2014). Unique features of the m6A methylome in *Arabidopsis thaliana*. Nat. Commun..

[12] Zhou J (2015). Dynamic m(6)A mRNA methylation directs translational control of heat shock response. Nature.

[13] Meyer KD (2015). 5’ UTR m(6)A promotes Cap-independent translation. Cell.

[14] Meyer KD, Jaffrey SR (2017). Rethinking m(6)A readers, writers, and erasers. Annu. Rev. Cell Dev. Biol..

[15] Shi H, Wei J, He C (2019). Where, when, and how: context-dependent functions of RNA methylation writers, readers, and erasers. Mol. Cell.

[16] Yang Y, Hsu PJ, Chen YS, Yang YG (2018). Dynamic transcriptomic m(6)A decoration: writers, erasers, readers and functions in RNA metabolism. Cell Res..

[17] Patil DP, Pickering BF, Jaffrey SR (2018). Reading m(6)A in the transcriptome: m(6)A-binding proteins. Trends Cell Biol..

[18] Jia G (2011). N6-methyladenosine in nuclear RNA is a major substrate of the obesity-associated FTO. Nat. Chem. Biol..

[19] Zheng G (2013). ALKBH5 is a mammalian RNA demethylase that impacts RNA metabolism and mouse fertility. Mol. Cell.

[20] Mauer J (2017). Reversible methylation of m(6)Am in the 5’ cap controls mRNA stability. Nature.

[21] Bokar JA, Rath-Shambaugh ME, Ludwiczak R, Narayan P, Rottman F (1994). Characterization and partial purification of mRNA N6-adenosine methyltransferase from HeLa cell nuclei. Internal mRNA methylation requires a multisubunit complex. J. Biol. Chem..

[22] Bokar JA, Shambaugh ME, Polayes D, Matera AG, Rottman FM (1997). Purification and cDNA cloning of the AdoMet-binding subunit of the human mRNA (N6-adenosine)-methyltransferase. RNA.

[23] Horiuchi K (2013). Identification of Wilms’ tumor 1-associating protein complex and its role in alternative splicing and the cell cycle. J. Biol. Chem..

[24] Hongay CF, Orr-Weaver TL (2011). Drosophila Inducer of MEiosis 4 (IME4) is required for Notch signaling during oogenesis. Proc. Natl Acad. Sci. USA.

[25] Liu J (2014). A METTL3-METTL14 complex mediates mammalian nuclear RNA N6-adenosine methylation. Nat. Chem. Biol..

[26] Wang Y (2014). N6-methyladenosine modification destabilizes developmental regulators in embryonic stem cells. Nat. Cell Biol..

[27] Zhong S (2008). MTA is an Arabidopsis messenger RNA adenosine methylase and interacts with a homolog of a sex-specific splicing factor. Plant Cell.

[28] Ping XL (2014). Mammalian WTAP is a regulatory subunit of the RNA N6-methyladenosine methyltransferase. Cell Res..

[29] Agarwala SD, Blitzblau HG, Hochwagen A, Fink GR (2012). RNA methylation by the MIS complex regulates a cell fate decision in yeast. PLoS Genet..

[30] Schwartz S (2014). Perturbation of m6A writers reveals two distinct classes of mRNA methylation at internal and 5’ sites. Cell Rep..

[31] Yue Y (2018). VIRMA mediates preferential m(6)A mRNA methylation in 3’UTR and near stop codon and associates with alternative polyadenylation. Cell Discov..

[32] Patil DP (2016). m(6)A RNA methylation promotes XIST-mediated transcriptional repression. Nature.

[33] Lence T (2016). m(6)A modulates neuronal functions and sex determination in Drosophila. Nature.

[34] Knuckles P (2018). Zc3h13/Flacc is required for adenosine methylation by bridging the mRNA-binding factor Rbm15/Spenito to the m(6)A machinery component Wtap/Fl(2)d. Genes Dev..

[35] Wen J (2018). Zc3h13 regulates nuclear RNA m(6)A methylation and mouse embryonic stem cell self-renewal. Mol. Cell.

[36] Guo J, Tang HW, Li J, Perrimon N, Yan D (2018). Xio is a component of the Drosophila sex determination pathway and RNA N(6)-methyladenosine methyltransferase complex. Proc. Natl Acad. Sci. USA.

[37] Ruzicka K (2017). Identification of factors required for m(6) A mRNA methylation in Arabidopsis reveals a role for the conserved E3 ubiquitin ligase HAKAI. N. Phytol..

[38] Granadino B, Campuzano S, Sanchez L (1990). The *Drosophila melanogaster* fl(2)d gene is needed for the female-specific splicing of Sex-lethal RNA. EMBO J..

[39] Hilfiker A, Nothiger R (1991). The temperature-sensitive mutation vir (ts)(virilizer) identifies a new gene involved in sex determination of Drosophila. Roux Arch. Dev. Biol..

[40] Yan D (2014). A regulatory network of Drosophila germline stem cell self-renewal. Dev. Cell.

[41] Yan D, Perrimon N (2015). spenito is required for sex determination in *Drosophila melanogaster*. Proc. Natl Acad. Sci. USA.

[42] Penn JK (2008). Functioning of the Drosophila Wilms’-tumor-1-associated protein homolog, Fl(2)d, in Sex-lethal-dependent alternative splicing. Genetics.

[43] Hilfiker A, Amrein H, Dubendorfer A, Schneiter R, Nothiger R (1995). The gene virilizer is required for female-specific splicing controlled by Sxl, the master gene for sexual development in Drosophila. Development.

[44] Penalva LO (2000). The Drosophila fl(2)d gene, required for female-specific splicing of Sxl and tra pre-mRNAs, encodes a novel nuclear protein with a HQ-rich domain. Genetics.

[45] Niessen M, Schneiter R, Nothiger R (2001). Molecular identification of virilizer, a gene required for the expression of the sex-determining gene Sex-lethal in *Drosophila melanogaster*. Genetics.

[46] Ortega A (2003). Biochemical function of female-lethal (2)D/Wilms’ tumor suppressor-1-associated proteins in alternative pre-mRNA splicing. J. Biol. Chem..

[47] Kan L (2017). The m(6)A pathway facilitates sex determination in Drosophila. Nat. Commun..

[48] Haussmann IU (2016). m(6)A potentiates Sxl alternative pre-mRNA splicing for robust Drosophila sex determination. Nature.

[49] Wan C (2015). Panorama of ancient metazoan macromolecular complexes. Nature.

[50] Fujita Y (2002). Hakai, a c-Cbl-like protein, ubiquitinates and induces endocytosis of the E-cadherin complex. Nat. Cell Biol..

[51] Aparicio LA, Valladares M, Blanco M, Alonso G, Figueroa A (2012). Biological influence of Hakai in cancer: a 10-year review. Cancer Metastasis Rev..

[52] Kaido M, Wada H, Shindo M, Hayashi S (2009). Essential requirement for RING finger E3 ubiquitin ligase Hakai in early embryonic development of Drosophila. Genes Cells.

[53] Guruharsha KG (2011). A protein complex network of *Drosophila melanogaster*. Cell.

[54] Graveley BR (2011). The developmental transcriptome of *Drosophila melanogaster*. Nature.

[55] Port F, Chen HM, Lee T, Bullock SL (2014). Optimized CRISPR/Cas tools for efficient germline and somatic genome engineering in Drosophila. Proc. Natl Acad. Sci. USA.

[56] Qiao HH (2018). An efficient and multiple target transgenic RNAi technique with low toxicity in Drosophila. Nat. Commun..

[57] Ren X (2013). Optimized gene editing technology for Drosophila melanogaster using germ line-specific Cas9. Proc. Natl Acad. Sci. USA.

[58] Bell LR, Horabin JI, Schedl P, Cline TW (1991). Positive autoregulation of Sex-lethal by alternative splicing maintains the female determined state in Drosophila. Cell.

[59] Johnson ML, Nagengast AA, Salz HK (2010). PPS, a large multidomain protein, functions with Sex-lethal to regulate alternative splicing in Drosophila. PLoS Genet..

[60] SchuLtt C, Hilfiker A, Nothiger R (1998). virilizer regulates Sex-lethal in the germline of *Drosophila melanogaster*. Development.

[61] Samuels ME (1994). RNA binding by Sxl proteins in vitro and in vivo. Mol. Cell Biol..

[62] Sakamoto H, Inoue K, Higuchi I, Ono Y, Shimura Y (1992). Control of Drosophila Sex-lethal pre-mRNA splicing by its own female-specific product. Nucleic Acids Res..

[63] Linder B (2015). Single-nucleotide-resolution mapping of m6A and m6Am throughout the transcriptome. Nat. Methods.

[64] Garcias Morales D, Reyes JL (2021). A birds’-eye view of the activity and specificity of the mRNA m(6) A methyltransferase complex. Wiley Interdiscip. Rev. RNA.

[65] Figueroa A (2009). Novel roles of Hakai in cell proliferation and oncogenesis. Mol. Biol. Cell.

[66] Hsu PJ, Shi H, He C (2017). Epitranscriptomic influences on development and disease. Genome Biol..

[67] Kan, L. et al. A neural m^6^A/Ythdf pathway is required for learning and memory in Drosophila. *Nat. Commun.* **12**, 1458 (2021). 10.1038/s41467-021-21537-1 PMC7935873 33674589

[68] Zhang H (2018). Genome-wide maps of ribosomal occupancy provide insights into adaptive evolution and regulatory roles of uORFs during Drosophila development. PLoS Biol..

[69] Salz HK, Erickson JW (2010). Sex determination in Drosophila: the view from the top. Fly.

[70] Dominissini D, Moshitch-Moshkovitz S, Salmon-Divon M, Amariglio N, Rechavi G (2013). Transcriptome-wide mapping of N(6)-methyladenosine by m(6)A-seq based on immunocapturing and massively parallel sequencing. Nat. Protoc..

[71] Molinie B, Giallourakis CC (2017). Genome-wide location analyses of N6-methyladenosine modifications (m(6)A-Seq). Methods Mol. Biol..

[72] Kim D, Langmead B, Salzberg SL (2015). HISAT: a fast spliced aligner with low memory requirements. Nat. Methods.

[73] Meng J (2014). A protocol for RNA methylation differential analysis with MeRIP-Seq data and exomePeak R/Bioconductor package. Methods.

[74] Heinz S (2010). Simple combinations of lineage-determining transcription factors prime cis-regulatory elements required for macrophage and B cell identities. Mol. Cell.

[75] Yu G, Wang LG, He QY (2015). ChIPseeker: an R/Bioconductor package for ChIP peak annotation, comparison and visualization. Bioinformatics.

[76] Pertea M (2015). StringTie enables improved reconstruction of a transcriptome from RNA-seq reads. Nat. Biotechnol..

[77] Robinson MD, McCarthy DJ, Smyth GK (2010). edgeR: a Bioconductor package for differential expression analysis of digital gene expression data. Bioinformatics.

[78] Shen S (2014). rMATS: robust and flexible detection of differential alternative splicing from replicate RNA-Seq data. Proc. Natl Acad. Sci. USA.

