# Supplementary material for: Role of Hakai in m6A modification pathway in Drosophila
Source: Nat Commun. 2021 Apr 12;12:2159. doi: 10.1038/s41467-021-22424-5 (PMC8041851; doi:10.1038/s41467-021-22424-5)
Supplement: Supplementary file 1 — Supplementary information [file 41467_2021_22424_MOESM1_ESM.pdf]

a

Our mass-spec data

Bait	Pulldown	Total Spectrum Count
GFP	Hakai	0
Fl(2)d	Hakai	5
Nito	Hakai	2

b

DPiM mass-spec data

Bait	Pulldown	Unique peptides	Total peptides
Hakai	Vir	5	6
Hakai	Fl(2)d	5	5
Hakai	Nito	3	3
Flacc	Hakai	3	3

Supplementary Figure 1. Interactions between Hakai and other m^{6}A writer components from Mass-spec data

a In our previous mass-spec experiments, using Fl(2)d and Nito, but not GFP, as baits can pull down Hakai. **b** From DPiM mass-spec dataset, Hakai as a bait can pull down Vir, Fl(2)d and Nito, while Flacc as a bait can pull down Hakai. Unique and total peptides pulled were shown.

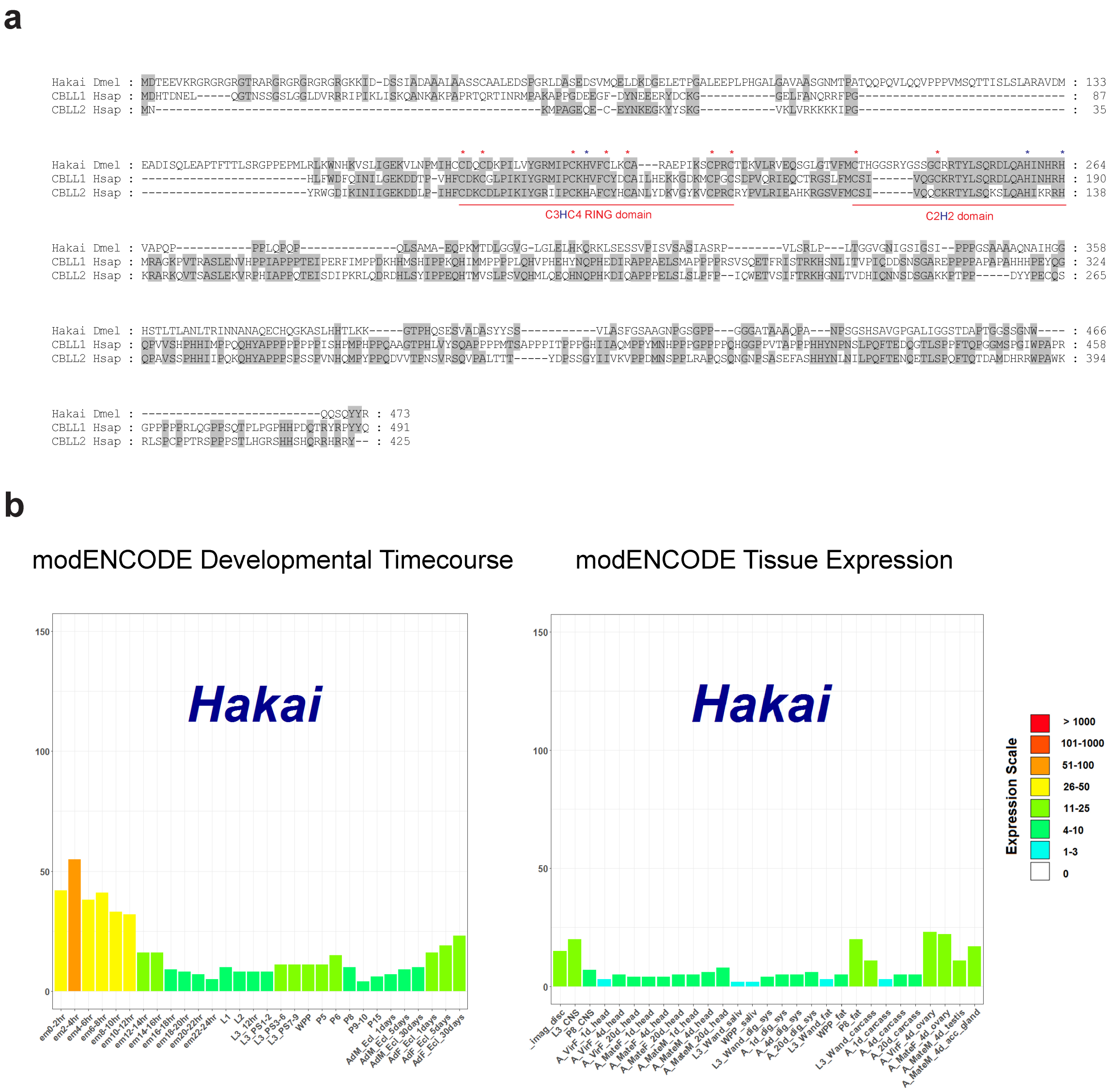

Supplementary Figure 2. Protein sequence and transcript expression pattern of *Hakai*

a Alignment of *Drosophila* Hakai and its homologs human CBLL1 and CBLL2 by COBALT. Identical residues are shaded. The conserved C3HC4-type RING domain and C2H2 zinc-finger domain are underlined in red. **b** RPKM measurements of *Hakai* from modENCODE developmental timecourse and tissue expression dataset.

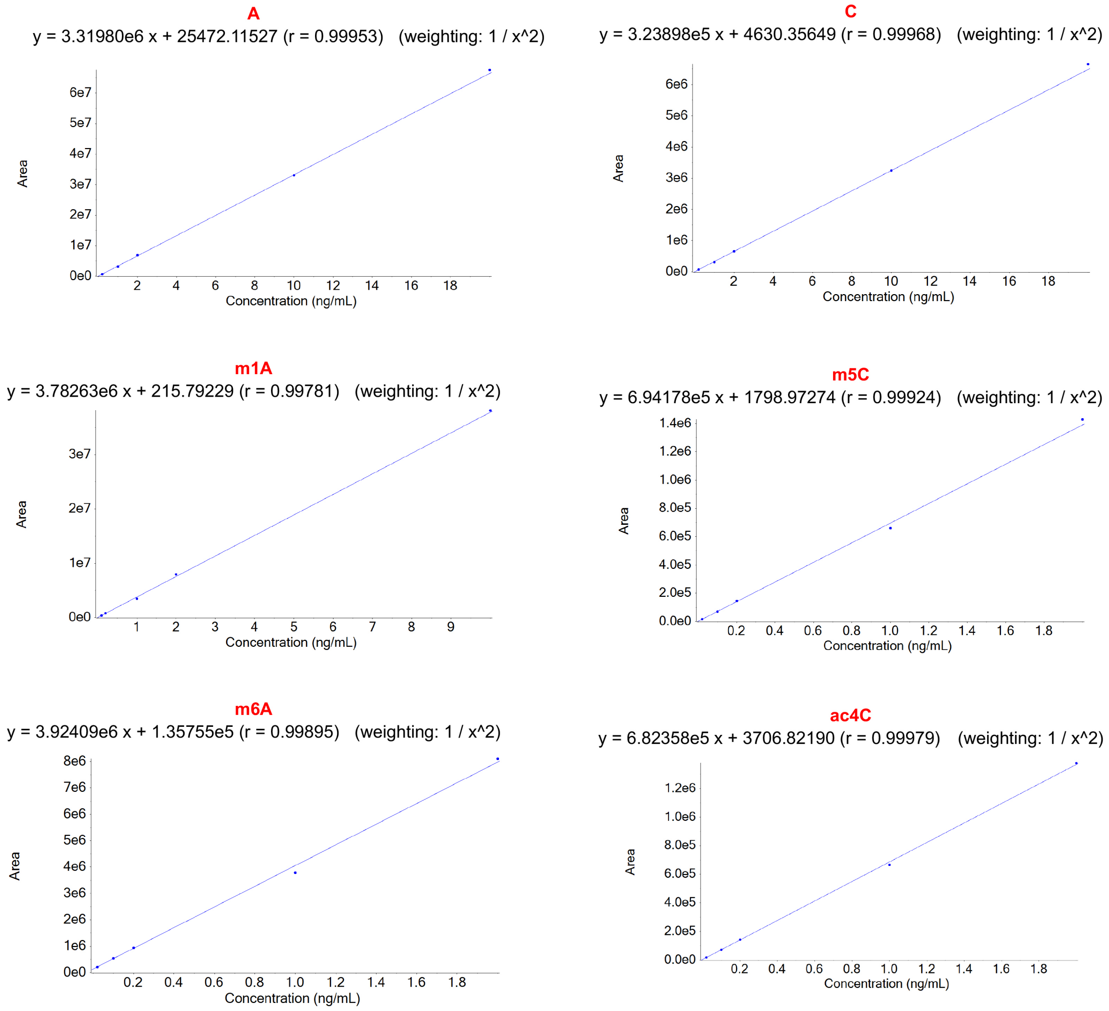

Supplementary Figure 3. Calibration curves for nucleoside quantification

Standard calibration curves used for absolute quantifications of adenosine (A), N6-methyladenosine (m^6A), N1-methyladenosine (m^1A), cytidine (C), 5-methylcytidine (m^5C), and N4-acetylcytidine (ac^4C).

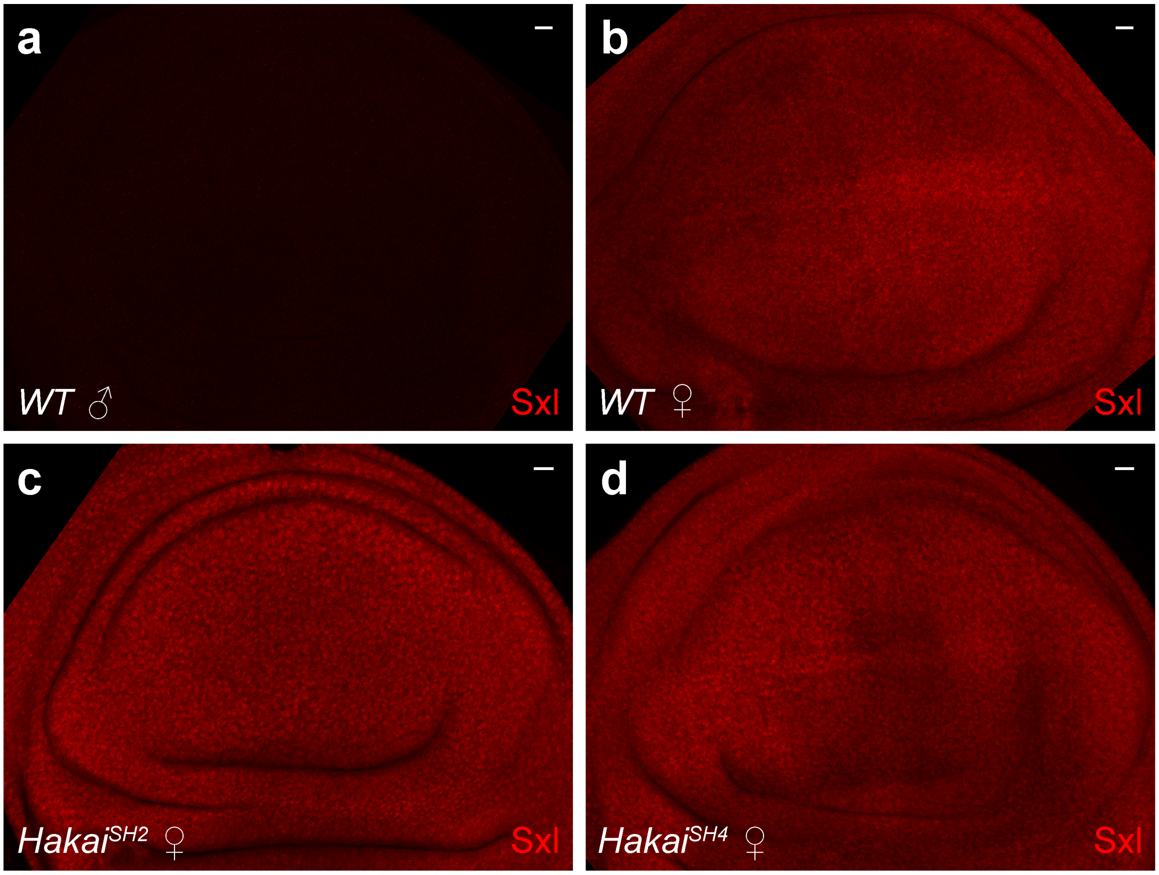

Supplementary Figure 4. Hakai is not required for Sxl protein levels in discs

In wild-type, Sxl protein is only expressed in female (**b**), but not male (**a**) wing discs, as shown by Sxl antibody staining. **c-d** Sxl protein levels are not reduced in $Hakai^{SH2}$ and $Hakai^{SH4}$ female discs. The experiments in **a-d** were repeated at least twice independently with similar results, and each time around 30 wing discs for any genotype were examined. Scale bars, 10 μm.

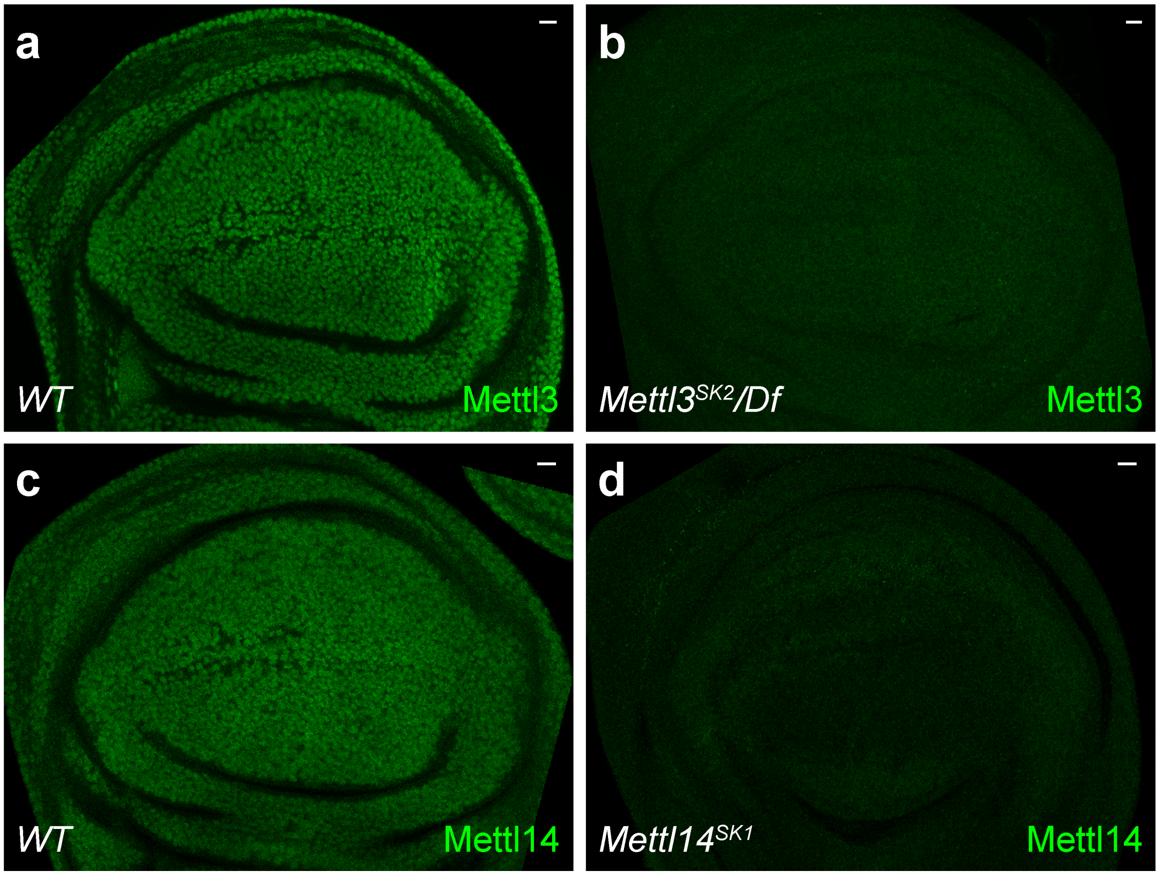

Supplementary Figure 5. Validation of Mettl3 and Mettl14 antibodies and alleles

In wild-type wing discs, Mettl3 (**a**) and Mettl14 (**c**) show ubiquitous nuclear staining. In *Mettl3*^{SK2}*/Df* or *Mettl14*^{SK1} mutant discs, no detectable level of Mettl3 (**b**) or Mettl14 (**d**) protein remains, respectively. The experiments in **a-d** were repeated at least twice independently with similar results, and each time around 30 wing discs for any genotype were examined. Scale bars, 10 μm.

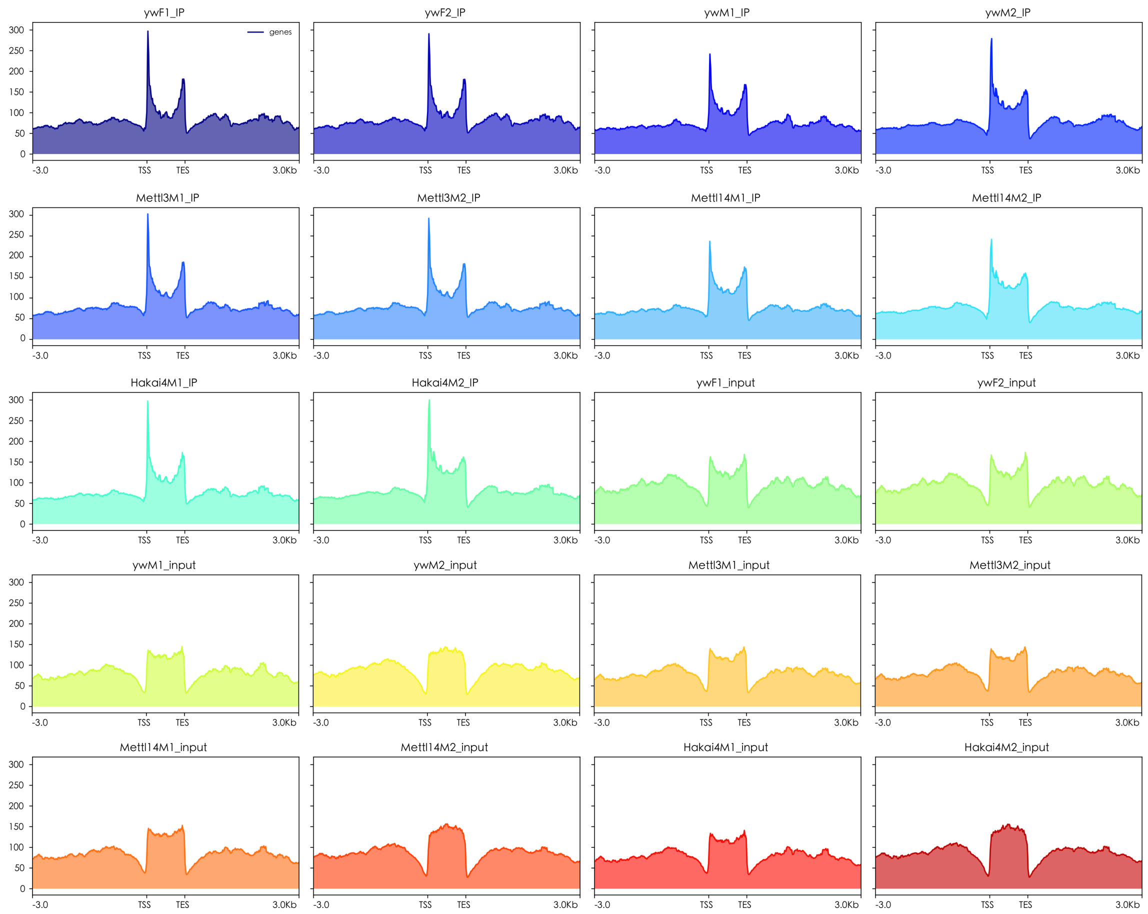

Supplementary Figure 6. Distribution of mapped reads in m^{6}A-IP and input samples

Distribution of mapped reads from m^{6}A-IP and input along genes for *yw* female, *yw* male, *Mettl3* male, *Mettl14* male, and *Hakai* male flies. We performed two replicates for each genotype. Note the enrichment around transcription start sites (TSS) and transcription end sites (TES) in IP samples but not input, validating our MeRIP-seq.

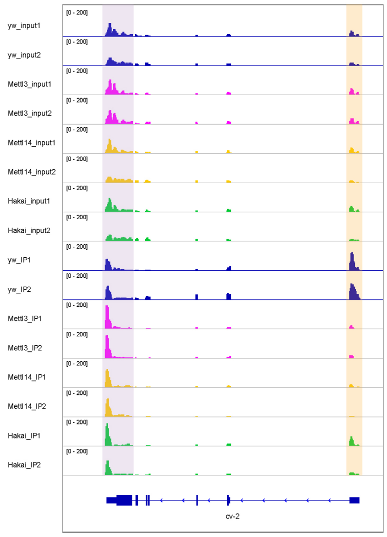
yw_input1
yw_input2
Mettl3_input1
Mettl3_input2
Mettl14_input1
Mettl14_input2
Hakai_input1
Hakai_input2
yw_IP1
yw_IP2
Mettl3_IP1
Mettl3_IP2
Mettl14_IP1
Mettl14_IP2
Hakai_IP1
Hakai_IP2
[0 - 200]
cv-2

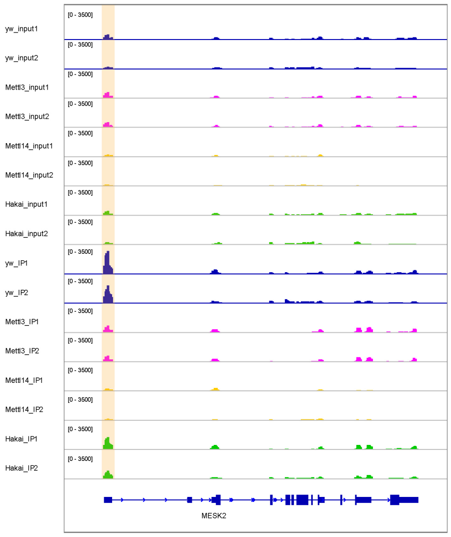
yw_input1
yw_input2
Mettl3_input1
Mettl3_input2
Mettl14_input1
Mettl14_input2
Hakai_input1
Hakai_input2
yw_IP1
yw_IP2
Mettl3_IP1
Mettl3_IP2
Mettl14_IP1
Mettl14_IP2
Hakai_IP1
Hakai_IP2
[0 - 3500]
MESK2

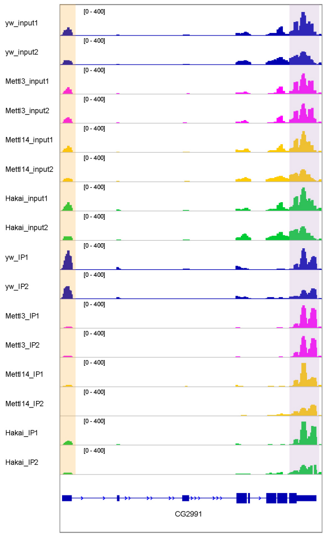
yw_input1
yw_input2
Mettl3_input1
Mettl3_input2
Mettl14_input1
Mettl14_input2
Hakai_input1
Hakai_input2
yw_IP1
yw_IP2
Mettl3_IP1
Mettl3_IP2
Mettl14_IP1
Mettl14_IP2
Hakai_IP1
Hakai_IP2
[0 - 400]
CG2991

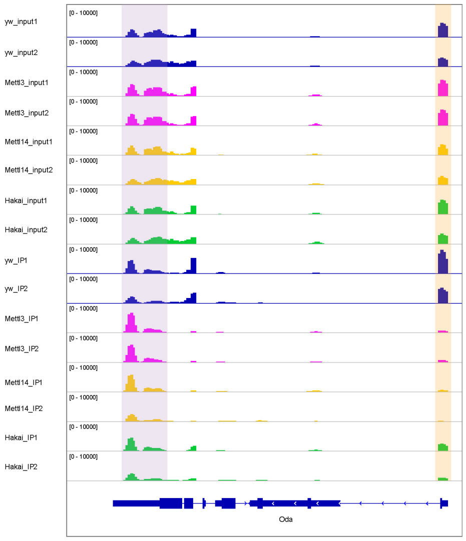
yw_input1
yw_input2
Mettl3_input1
Mettl3_input2
Mettl14_input1
Mettl14_input2
Hakai_input1
Hakai_input2
yw_IP1
yw_IP2
Mettl3_IP1
Mettl3_IP2
Mettl14_IP1
Mettl14_IP2
Hakai_IP1
Hakai_IP2
[0 - 10000]
Oda

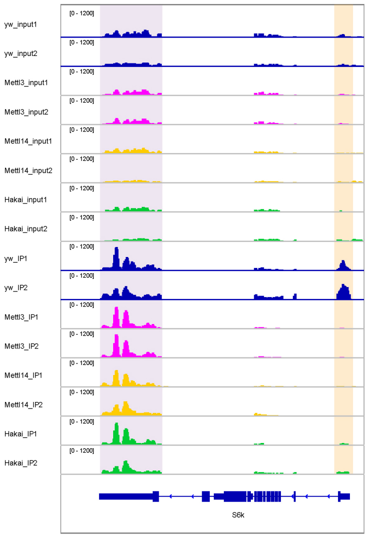
yw_input1
yw_input2
Mettl3_input1
Mettl3_input2
Mettl14_input1
Mettl14_input2
Hakai_input1
Hakai_input2
yw_IP1
yw_IP2
Mettl3_IP1
Mettl3_IP2
Mettl14_IP1
Mettl14_IP2
Hakai_IP1
Hakai_IP2
[0 - 1200]
S6k

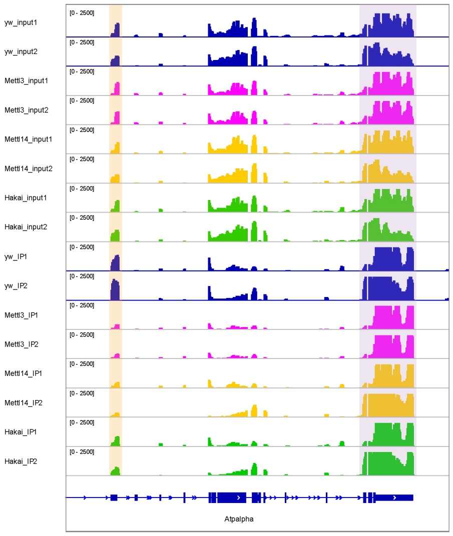
yw_input1
yw_input2
Mettl3_input1
Mettl3_input2
Mettl14_input1
Mettl14_input2
Hakai_input1
Hakai_input2
yw_IP1
yw_IP2
Mettl3_IP1
Mettl3_IP2
Mettl14_IP1
Mettl14_IP2
Hakai_IP1
Hakai_IP2
[0 - 2500]
Atpalpha

Supplementary Figure 7. Examples of reduced m^{6}A peaks in *Mettl3*, *Mettl14*, and *Hakai* mutants

Integrative Genomics Viewer (IGV) tracks displaying MeRIP-seq (lower panels, IP) and RNA-seq (upper panels, input) reads along indicated mRNAs in *yw*, *Mettl3*, *Mettl14*, and *Hakai* male flies. Two replicates are shown. Note the reduced peaks in 5' UTRs (shaded in yellow) and peaks in 3' UTRs (shaded in purple) are generally not changed.

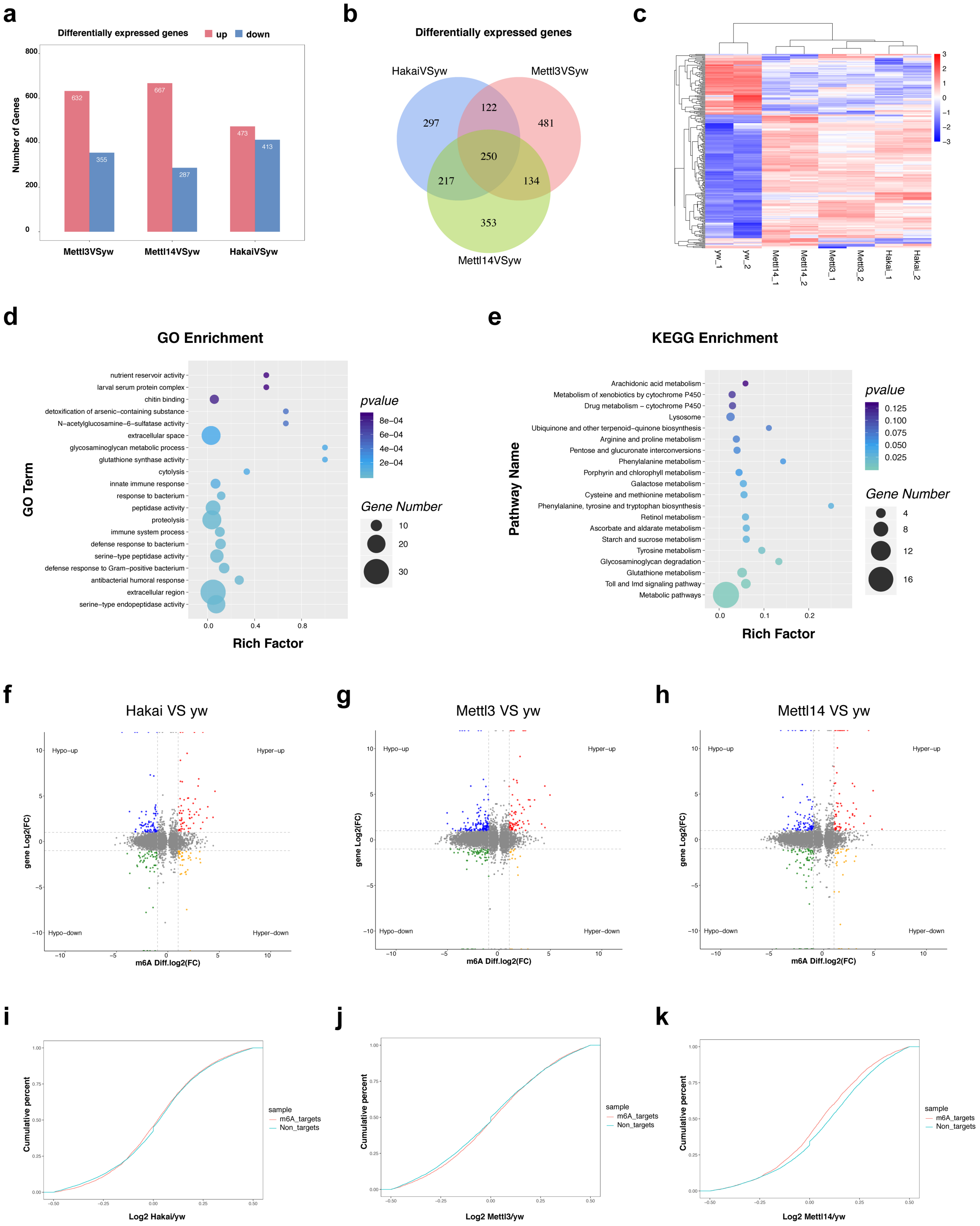

Supplementary Figure 8. Hakai regulates common transcripts with Mettl3 and Mettl14

a Numbers of differentially expressed genes ($p<0.05$ and fold change ≥ 2 or ≤ 0.5) in *Mettl3*, *Mettl14*, and *Hakai* mutant male adult flies versus *yw* control. **b** Venn diagram showing common differentially expressed genes between them. **c** Heat map showing the relative expression of 250 common differentially expressed genes in *Mettl3*, *Mettl14*, *Hakai* and *yw* flies. **d** GO term and **e** KEGG pathway analysis of 250 common differentially expressed genes between *Mettl3*, *Mettl14*, and *Hakai.* The top 20 terms are displayed. Two-sided Fisher's exact test. **f-h** Four quadrant plots showing differential m^6A peaks together with differential mRNA expression in *Mettl3* (**g**), *Mettl14* (**h**), and *Hakai* (**f**) mutants versus *yw*. Hyper- or Hypo- means increased or decreased m^6A peaks; Up or down means increased or decreased mRNA expression levels. **i-k** Cumulative percent distribution of fold changes (log2) of RNA expression in *Mettl3* (**j**), *Mettl14* (**k**), and *Hakai* (**i**) mutants over *yw* separated between m^6A targets and non-targets.

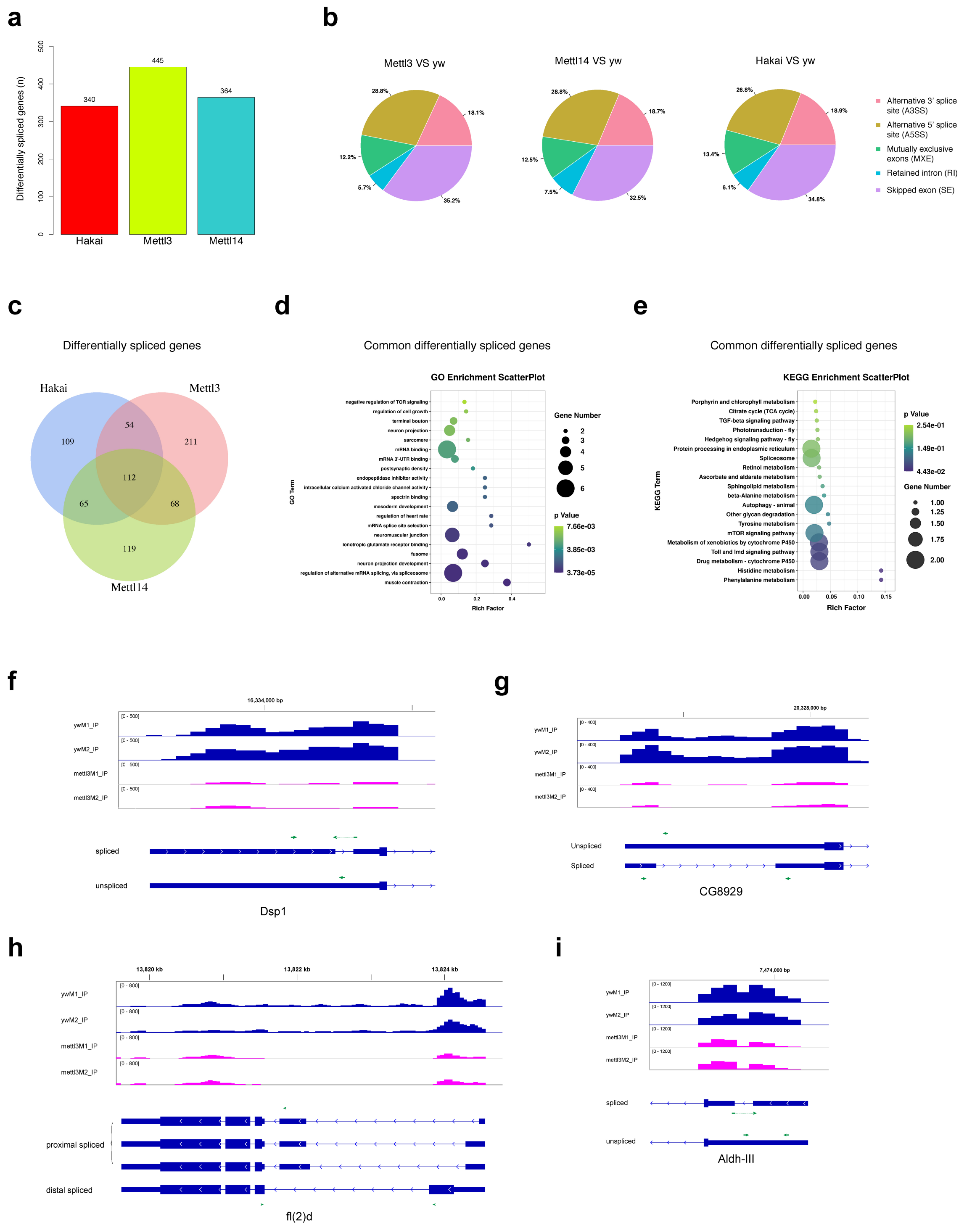

Supplementary Figure 9. Splicing analysis in *Mettl3*, *Mettl14*, or *Hakai* mutant

a Numbers of differentially spliced genes (FDR<0.05 and IncLevelDifference≥0.2 or ≤-0.2) in *Mettl3*, *Mettl14*, and *Hakai* mutant male adult flies versus *yw* control. **b**

Classification of differentially spliced events in the three mutants. **c** Venn diagram showing common differentially spliced genes between them. **d** GO term and **e** KEGG pathway analysis of common differentially spliced genes between *Mettl3*, *Mettl14*, and *Hakai.* The top 20 terms are displayed. Two-sided Fisher's exact test. **f-i** IGV tracks displaying MeRIP-seq along four m^6A-dependent, alternatively spliced regions in *yw* and *Mettl3* mutant flies. The heavy methylated m^6A peaks around splicing junctions in wild-type are reduced in *Mettl3* mutant. The positions of qPCR primers used in Fig. 3c are also indicated.
