# Supplementary material for: Role of Hakai in m6A modification pathway in Drosophila
Source: Nat Commun. 2021 Apr 12;12:2159. doi: 10.1038/s41467-021-22424-5 (PMC8041851; doi:10.1038/s41467-021-22424-5)
Supplement: Supplementary file 12 — Description of additional supplementary files [file 41467_2021_22424_MOESM12_ESM.docx]

Description of additional supplementary information

Title: Supplementary Data 1.

Description: m6A peak calling in yw, Mettl3, Mettl14 and Hakai male adult flies and yw female adult flies Peaks were called by R package exomepeak and p-value calculated by the ctest function.

Title: Supplementary Data 2.

Description: Differential m6A peaks in Mettl3, Mettl14 and Hakai male adult flies versus yw Diffpeaks were analyzed by R package exomepeak and p-value calculated by the bltest function.

Title: Supplementary Data 3.

Description: Gene expression in yw, Mettl3, Mettl14 and Hakai male adult flies and yw female adult flies analyzed by RNA-seq

Title: Supplementary Data 4.

Description: Differential gene expression in Mettl3, Mettl14 and Hakai male adult flies versus yw Differential gene expression and statistical analysis were performed by R package edgeR.

Title: Supplementary Data 5.

Description: Differential m6A peaks and differential gene expression in Mettl3, Mettl14 and Hakai male adult flies versus yw

Title: Supplementary Data 6.

Description: Differential spliced events categorized by A3SS, A5SS, MXE, RI, and SE in Mettl3 male adult flies versus yw Data S6-8, differential splicing and statistical analysis were performed by rMATS software.

Title: Supplementary Data 7.

Description: Differential spliced events categorized by A3SS, A5SS, MXE, RI, and SE in Mettl14 male adult flies versus yw

Title: Supplementary Data 8.

Description: Differential spliced events categorized by A3SS, A5SS, MXE, RI, and SE in Hakai male adult flies versus yw

Title: Supplementary Data 9.

Description: Sequences of all primers used in this study
