# Supplementary material for: Role of Hakai in m6A modification pathway in Drosophila
Source: Nat Commun. 2021 Apr 12;12:2159. doi: 10.1038/s41467-021-22424-5 (PMC8041851; doi:10.1038/s41467-021-22424-5)
Supplement: Supplementary file 13 — Reporting Summary [file 41467_2021_22424_MOESM13_ESM.pdf]

nature research

Corresponding author(s): Dong Yan

Last updated by author(s): Mar 2, 2021

Reporting Summary

Nature Research wishes to improve the reproducibility of the work that we publish. This form provides structure for consistency and transparency in reporting. For further information on Nature Research policies, see our Editorial Policies and the Editorial Policy Checklist.

Statistics

For all statistical analyses, confirm that the following items are present in the figure legend, table legend, main text, or Methods section.

n/a	Confirmed	
☐	☒	The exact sample size (*n*) for each experimental group/condition, given as a discrete number and unit of measurement
☐	☒	A statement on whether measurements were taken from distinct samples or whether the same sample was measured repeatedly
☐	☒	The statistical test(s) used AND whether they are one- or two-sided<br>*Only common tests should be described solely by name; describe more complex techniques in the Methods section.*
☒	☐	A description of all covariates tested
☒	☐	A description of any assumptions or corrections, such as tests of normality and adjustment for multiple comparisons
☐	☒	A full description of the statistical parameters including central tendency (e.g. means) or other basic estimates (e.g. regression coefficient) AND variation (e.g. standard deviation) or associated estimates of uncertainty (e.g. confidence intervals)
☐	☒	For null hypothesis testing, the test statistic (e.g. *F*, *t*, *r*) with confidence intervals, effect sizes, degrees of freedom and *P* value noted<br>*Give P values as exact values whenever suitable.*
☒	☐	For Bayesian analysis, information on the choice of priors and Markov chain Monte Carlo settings
☒	☐	For hierarchical and complex designs, identification of the appropriate level for tests and full reporting of outcomes
☒	☐	Estimates of effect sizes (e.g. Cohen's *d*, Pearson's *r*), indicating how they were calculated

Our web collection on statistics for biologists contains articles on many of the points above.

Software and code

Policy information about availability of computer code

Data collection: Zeiss LSM880 was used for microscopy image acquisition.

Data analysis: Statistical analysis was performed using GraphPad Prism (v9.0.0).
fastp (v0.19.4)
HISAT2 (v2.0.4)
exomePeak (v1.9.1)
HOMER (v4.1)
ChIPseeker (v1.18.0)
StringTie (v1.3.4)
edgeR (v4.1)
rMATS (v4.0.1)

For manuscripts utilizing custom algorithms or software that are central to the research but not yet described in published literature, software must be made available to editors and reviewers. We strongly encourage code deposition in a community repository (e.g. GitHub). See the Nature Research guidelines for submitting code & software for further information.

Data

Policy information about availability of data

All manuscripts must include a data availability statement. This statement should provide the following information, where applicable:

- Accession codes, unique identifiers, or web links for publicly available datasets
- A list of figures that have associated raw data
- A description of any restrictions on data availability

High-throughput m6A-seq data were deposited into the GEO database with accession number:GSE155662. It includes all raw files, peak text files, FPKM value text files, and bigWig files for visualization.

Field-specific reporting

Please select the one below that is the best fit for your research. If you are not sure, read the appropriate sections before making your selection.

☒ Life sciences ☐ Behavioural & social sciences ☐ Ecological, evolutionary & environmental sciences

For a reference copy of the document with all sections, see nature.com/documents/nr-reporting-summary-flat.pdf

Life sciences study design

All studies must disclose on these points even when the disclosure is negative.

Sample size	No statistical methods were used to predetermine sample size. Sample sizes were chosen based on previous experience and similar experiments in the literature. Sample sizes were indicated in the figure legends and Methods.
Data exclusions	No data were excluded from the analysis.
Replication	Repeat numbers of the experiments were indicated in the figure legends. All attempts at replication were successful.
Randomization	Samples were randomly assigned.
Blinding	The experiments were not blinded as the obvious mutant phenotypes make blinding surplus.

Reporting for specific materials, systems and methods

We require information from authors about some types of materials, experimental systems and methods used in many studies. Here, indicate whether each material, system or method listed is relevant to your study. If you are not sure if a list item applies to your research, read the appropriate section before selecting a response.

Materials & experimental systems

n/a	Involved in the study	
☐	☒	Antibodies
☐	☒	Eukaryotic cell lines
☒	☐	Palaeontology and archaeology
☐	☒	Animals and other organisms
☒	☐	Human research participants
☒	☐	Clinical data
☒	☐	Dual use research of concern

Methods

n/a	Involved in the study	
☒	☐	ChIP-seq
☒	☐	Flow cytometry
☒	☐	MRI-based neuroimaging

Antibodies

Antibodies used	mouse anti-Fl(2)d (9G2, DSHB), rat anti-Ecad (DCAD2, DSHB), mouse anti-Sxl (M18, DSHB), rabbit anti-Flacc (self-made), rabbit anti-Hakai (self-made), rabbit anti-Vir (self-made), rabbit anti-Mettl3 (self-made), rabbit anti-Mettl14 (self-made), rabbit anti-GFP (A6455, Molecular Probes), rat anti-HA (3F10, Roche), rabbit anti-m6A (202003, Synaptic Systems), Donkey anti-Mouse IgG (H+L) (Alexa Fluor 488) (Thermo Fisher Scientific, A21202), Donkey anti-Rabbit IgG (H+L) (Alexa Fluor 488) (Thermo Fisher Scientific, A21206), Cy3-conjugated Donkey anti-Mouse IgG (H+L) (Jackson ImmunoResearch, 715-165-150), Cy3-conjugated Donkey anti-Rabbit IgG (H+L) (Jackson ImmunoResearch, 711-165-152). The concentrations used were described in the methods section.
Validation	Hakai antibody was validated by immunostaining in RNAi, CRISPR and mutant wing discs (Fig. 2d-e, 4g, 4f').<br>Vir antibody was validated by immunostaining in RNAi wing discs (Fig. 4m).<br>Mettl3 and Mettl14 antibodies were validated by immunostaining in mutant wing discs (Fig. S5a-d).<br>rabbit anti-Flacc (self-made) was validated previously in: Guo, J et al. PNAS, 115(14):3674-3679 (2018).<br>mouse anti-Fl(2)d (9G2, DSHB), rat anti-Ecad (DCAD2, DSHB), and mouse anti-Sxl (M18, DSHB) were obtained from Developmental

Studies Hybridoma Bank and their information are described in https://dshb.biology.uiowa.edu.
rabbit anti-GFP (A6455, Molecular Probes), rat anti-HA (3F10, Roche), and rabbit anti-m6A (202003, Synaptic Systems) are widely-used commercial antibodies and their information are described in the companies' websites.

Eukaryotic cell lines

Policy information about cell lines

Cell line source(s)	Drosophila S2 cells were kindly provided by Prof Yongzhen Xu at Wuhan University as described in: Li, L et al. Nat. Commun. 11:5608 (2020). HeLa cells were purchased from the Cell Bank of the Chinese Academy of Sciences (Shanghai, China).
Authentication	None
Mycoplasma contamination	Not tested for mycoplasma contamination
Commonly misidentified lines (See ICLAC register)	None

Animals and other organisms

Policy information about studies involving animals; ARRIVE guidelines recommended for reporting animal research

Laboratory animals	Drosophila Melanogaster of the following genotypes were used:<br>w1118<br>yw<br>Mettl3SK2<br>Mettl14SK1<br>Df(3R)Exel6197<br>ap-Gal4<br>Hakai shRNA (VDRC 330548)<br>vir shRNA (HMC03908, Bloomington 55694)<br>fl(2)d shRNA (HMC03833, Bloomington 55674)<br>flacc shRNA (VDRC 35212GD)<br>actin-Cas9 (Bloomington 54590)<br>nanos-Cas9 (Bloomington 78782)<br>U6-Hakai-sgRNA<br>HakaiSH2<br>HakaiSH4<br>Hakai shRNA (TH14422.S)<br>Hakai shRNA (TH14423.S)<br>Immunostaining was performed on 3rd instar larval wing discs. Adult flies used in the paper were about 1-2 days old; the sex of these flies were indicated in Fig. 3b, Fig. 6d-g, Fig. S4, Fig. 2 legend, Fig. 3 legend, Fig. 6 legend, and Fig. 7 legend.
Wild animals	The study did not involve wild animals.
Field-collected samples	The study did not involve field-collected samples.
Ethics oversight	Not applicable.

Note that full information on the approval of the study protocol must also be provided in the manuscript.
